# High Resolution Imaging of Nonequilibrium Colloidal Self-Assembly via Photofixation

**DOI:** 10.1021/acsnano.5c22002

**Published:** 2026-02-12

**Authors:** Jagannath Satpathy, Jim Jui-Kai Chen, Gang Wen, Hiroshi Masuhara, Sudipta Seth, Volker Leen, Susana Rocha, Johan Hofkens, Boris Louis, Roger Bresolí-Obach

**Affiliations:** † Laboratory for Photochemistry and Spectroscopy, Division for Molecular Imaging and Photonics, Department of Chemistry, 26657 KU Leuven, Leuven 3001, Belgium; ‡ Department of Biotechnology and Biophysics, Biocenter, 9190 University of Würzburg, Am Hubland, 97074 Würzburg, Germany; § Department of Applied Chemistry and Center for Emergent Functional Matter Science, 34914 National Yang Ming Chiao Tung University, Hsinchu 300093, Taiwan; ∥ AppLightChem, Department of Analytical and Applied Chemistry, Institut Químic de Sarrià, Universitat Ramon Llull, Via Augusta 390, Barcelona 08017, Spain; ⊥ Chrometra Scientific B.V., Merelnest 3, 3470 Kortenaken, Belgium; # Max Planck Institute for Polymer Research, 55128 Mainz, Germany

**Keywords:** colloidal self-assembly, optical matter, optical trapping, photopolymerization, 3D imaging, scanning electron microscopy (SEM), STED microscopy

## Abstract

The self-organization of colloidal nanoparticles into complex structures, both in equilibrium and out-of-equilibrium, is a growing area in colloidal science with potential for creating functional materials. While equilibrium assemblies form stable and periodic structures, out-of-equilibrium (or active) assemblies exhibit dynamic, reconfigurable behavior under external stimuli. Therefore, understanding the structure–function relationships in these assemblies remains challenging due to their transient nature and limitations of current characterization methods. In this work, we present a methodology termed Fixation and Resolving of Colloidal Active Matter Ensembles (FRAME). FRAME combines UV photopolymerization to fix nonequilibrium colloidal assemblies with high-resolution imaging techniques, including 3D confocal microscopy, SEM and 3D STED super-resolution imaging, for subsequent structural characterization. We applied this method to Optical Matter (OM) structures formed within an optical trap at the glass/water interface. Using FRAME, we conducted a detailed analysis of OM structures composed of colloidal nanoparticles ranging from 200 nm to 1 μm. We demonstrate the robustness of this method by validating that the fixation process does not alter structural properties, allowing for accurate structural analysis. FRAME offers a distinct approach for investigating nonequilibrium colloidal assemblies, enabling the way for their rational design and application across a broad range of colloidal systems.

## Graphical abstract

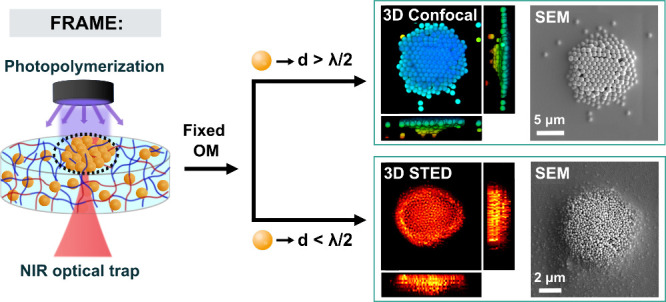

## 1 Introduction

The self-organization of colloidal nanoparticles (gold, silica, polystyrene, ...) into intricate and/or periodic structures is crucial due to their potential to create functional materials.^1,2^ Colloidal self-assemblies fall into equilibrium (passive) and out-of-equilibrium (active) types. Equilibrium assemblies form thermodynamically stable, highly periodic structures that can be used in functional materials like colloidal photonic crystals,^3,4^ metamaterials exhibiting high (*n* > 3) or negative refractive indices,^5–14^ and optoelectronic metamaterials based on quantum dot superlattices showing superfluorescence.^15,16^ Conversely, out-of-equilibrium, or active, self-assemblies respond dynamically to external stimuli (chemical, electrical, optical, magnetic, etc.), offer the advantage of being reconfigurable^2^ and are promising model systems for naturally occurring system including flocking and swarming, with applications in colloidal robotics.^17–20^ In both cases, understanding the interactions between the individual components is essential for controlling their spatial arrangement, which determines their properties.

Due to their dynamic nature, nonequilibrium assemblies of nanoparticles are inherently more challenging to investigate.^2,20–26^ Indeed, because of their transient behavior, both the assembling and monitoring of their properties need to be done simultaneously using a single experimental setup. This increases complexity of the setup and limits the range of applicable techniques. Furthermore, their dynamics restrict the number of available methods, as even optical microscopy struggles to distinguish individual particles in dense, 3D-packed assemblies of small (below the diffraction limit) particles.^27–29^ Therefore, alternative approaches are needed to study the structure, geometry, and properties of nonequilibrium colloidal assemblies.

Several dynamic and static approaches have been applied to study nonequilibrium colloidal assemblies. Dynamic approaches, including single-particle tracking (SPT) analysis, are used to understand the behavior of these assemblies in real time.^26,27,30,31^ However, SPT analysis is unable to track particles when the number of particles in the assembly increases, particularly if these particles are at or below the diffraction limit of optical microscopy, due to overlapping trajectories and resolution constraints. Alternatively, nonequilibrium colloidal assemblies can be studied by using static approaches like photopolymerization,^32–34^ optothermal manipulation,^35,36^ and light-triggered assembly fixation.^37–40^

While photopolymerization enables in situ immobilization of nanoparticle assemblies, it remains challenging to fix optical matter (OM) structures containing a large number of nanoparticles without distortion of order.^32^ Other approaches, such as thermophoresis-induced polymer collapse, drive nanoparticle aggregation under localized light heating and enable interface-confined assembly. However, the required heating can disturb the ordering of the resulting structures and offers only limited spatial and dynamic control over the assembly process.^33^ Additionally, opto-thermophoretic assembly in hydrogels has not demonstrated the fixation of assemblies containing a large number of particles or small-sized nanoparticles, and control over interparticle arrangement remains limited in such systems.^34^ Related photopolymerization strategies in colloidal photonic crystals and hydrogel-based photonic devices mainly focus on locking in preassembled or near-equilibrium structures,^41–43^ rather than transient optically bound OM. For example, UV-cross-linking in polyacrylamide hydrogels has been used to immobilize highly charged colloidal crystals, preserving preassembled 3D mesostructured order for subsequent templating/processing.^44^ These photopolymerization, optothermal and light-triggered fixation methods therefore remain insufficient for fixing large nonequilibrium, optically bound transient colloidal assemblies under mild, well-controlled gelation conditions that preserve the original optical binding behavior, and they provide only limited information on the gelation process. In addition, prior approaches generally lack validated fixation fidelity, high-resolution structural read-out (high-resolution SEM and 3D STED for subdiffraction assemblies), postfixation optical characterization (e.g., dark-field scattering for metamaterial relevance), and long-term structural stability.

To address these challenges, we present a workflow termed: Fixation and Resolving of Colloidal Active Matter Ensembles (FRAME), designed to investigate transient and nonequilibrium colloidal assemblies through a two-step process. First, UV photopolymerization is employed to permanently fix the nonequilibrium colloidal assembly within a hydrogel polymer network, thus capturing a snapshot of the dynamic assembly. Second, high-resolution imaging techniques including 3D confocal microscopy, SEM and STED super-resolution Z-stack imaging are employed to accurately characterize the structure and arrangement of the fixed assemblies. Validation through confocal and SEM imaging confirmed that the FRAME workflow does not alter the structural properties of the assemblies. While FRAME is currently demonstrated with 3D confocal microscopy, STED and SEM, this method can be extended to other high-resolution imaging techniques and spectroscopic techniques, offering additional opportunities to explore nonequilibrium assemblies. Importantly, FRAME enables the combination of multiple measurements on the same sample without requiring all modalities in a single setup.

Here, we demonstrated FRAME capabilities on OM, an example of nonequilibrium colloidal self-assembly. In OM nanoparticles self-organize through light-matter interactions, referred to as optical bonds, resulting in periodic arrangements.^27,45–49^ These optical bonds originate from multiple scattering interactions between these particles. As a consequence, OMs can be engineered and reconfigured “on-the-fly” by modifying the input light properties (wavelength, polarization, intensity profile, ...).^50,51^ Many examples of OM can be found in literature, including dumbbell-shaped assemblies of gold particles, chain-like structures of silver particles, and hexagonal clusters of polystyrene particles.^52–54^ These assemblies have also been shown to extend outside the irradiated area, a phenomenon still poorly understood.^27,55^ However, detailed information on the structure and/or their properties is often missing, particularly when the particle density increases, or their size decreases. OM composed by small particles are of special interest, as they are small enough to satisfy the Rayleigh scattering criteria, facilitating the comparison between theoretical models and experimental observations, an essential step toward unravelling the phenomenon.^45,51^ Moreover, the study of OM formed from subdiffraction-limit particles opens pathways toward the formation of colloidal metamaterials with tunable refractive index properties. In this context, we applied FRAME to study OM composed of particles ranging from 200 nm (subdiffraction limit particles) to 1 μm, formed by optical trapping at the interface. For particles larger than the diffraction limit, FRAME enables full 3D structural analysis, including crystallinity and lattice parameters. For subdiffraction particles, high-resolution SEM resolves the surface structure but not the internal 3D order. To overcome this, we perform 3D STED imaging on FRAME-fixed assemblies, reconstructing a 3D volume that resolves individual 200 nm PSNPs and enables quantitative in-volume ordering analysis. Moreover, by providing direct high-resolution structural feedback, we show that FRAME enabled us to identify key parameters (e.g., laser focus) for the control of the crystal structure of the OM (concentric, hexagonal close packing, cubic close packing). Finally, FRAME enables us to make the OM (or any colloidal self-assembly) permanent, allowing it to be used in desired applications. Therefore, FRAME enables the way for the characterization and rational design of nonequilibrium colloidal self-assembly into functional materials.

## 2 Results and Discussion

### 2.1 Fixation of Nonequilibrium Colloidal Self-Assembly via UV-Photopolymerization

FRAME’s first step is the fixation of colloidal self-assemblies and needs to fit three essential criteria: (i) the immobilization process must not disrupt the self-assembly and maintain the sample’s optical transparency for observation; (ii) the photopolymerization reaction should be fast to prevent structural changes; and (iii) the gel mesh size must be small enough to ensure the fixation of the particles across a wide range of sizes.

The formation of a Polyacrylamide (PAA) hydrogel through UV-photopolymerization fits these criteria. Indeed, PAA exhibits good hydrophilicity, tunable mechanical properties and optical transparency.^56–58^ The reaction is initiated using lithium phenyl (2,4,6-trimethylbenzoyl) phosphinate (LAP) as a radical photoinitiator under 365 nm LED irradiation.^59^ LAP is combined with acrylamide (monomer) and bis­(acrylamide) (cross-linker) in Milli-Q (MQ) water to create a Polyacrylamide Photocuring Medium (PPM). The concentration of each component is optimized (12.5 wt % acrylamide, 0.3 wt % bis­(acrylamide), and 0.44 wt % LAP photoinitiator) to meet the requirements for effective polymerization and to respect the aforementioned conditions. To achieve this, we needed a compromise: the photoinitiator concentration had to be high enough for fast photopolymerization, yet low enough to minimize changes in the suspension’s physicochemical properties (e.g., viscosity, refractive index, and colloidal stability), thereby avoiding any perturbation of the original optical binding behavior. The final PPM allows polymerization within 0.2–2 s under optimized UV power irradiation of 25 mW/cm^2^ (detailed in Materials and Methods). Indeed, increasing the power density over 25 mW/cm^2^ did not further accelerate the polymerization rate and, in contrast, often led to nonuniform gel formation. Although increasing the irradiation intensity initially accelerates the polymerization rate, at very high power densities the recombination of radicals becomes significant, leading to a plateau or even decrease in polymerization efficiency.^60–62^ Moreover, high-power UV irradiation can induce localized thermal fluctuations, and the resulting temperature rise may perturb the stability of OM assemblies, potentially affecting their structural integrity during fixation. This formulation maintains a viscosity of 1.2 cP, close to that of water (1 cP), and features a gel mesh size small enough to immobilize 23 nm particles effectively, as validated by 3D SPT (see Section S1). The refractive index (RI) of water was measured to be 1.334, while the refractive indices of the PPM solution before and after gelation were 1.351 and 1.356, respectively.

To evaluate the developed FRAME workflow, OM consisting of 1 μm polystyrene microparticles (PSMPs) is chosen as a standard example of a nonequilibrium colloidal self-assembly. For this, the PSMPs are dispersed in the PPM. Then, the OM structure is formed by irradiating for 5 min with a focused 1064 nm laser beam at the glass/solution interface (Figure 1a) and subsequently fixed by turning the UV-LED on, before turning off the 1064 nm laser. Control experiments on 1 μm PSMPs assemblies in water, water under UV, and in PPM show that PPM does not significantly affect the optical trap induced assembly (Section S2).

**Figure 1:**
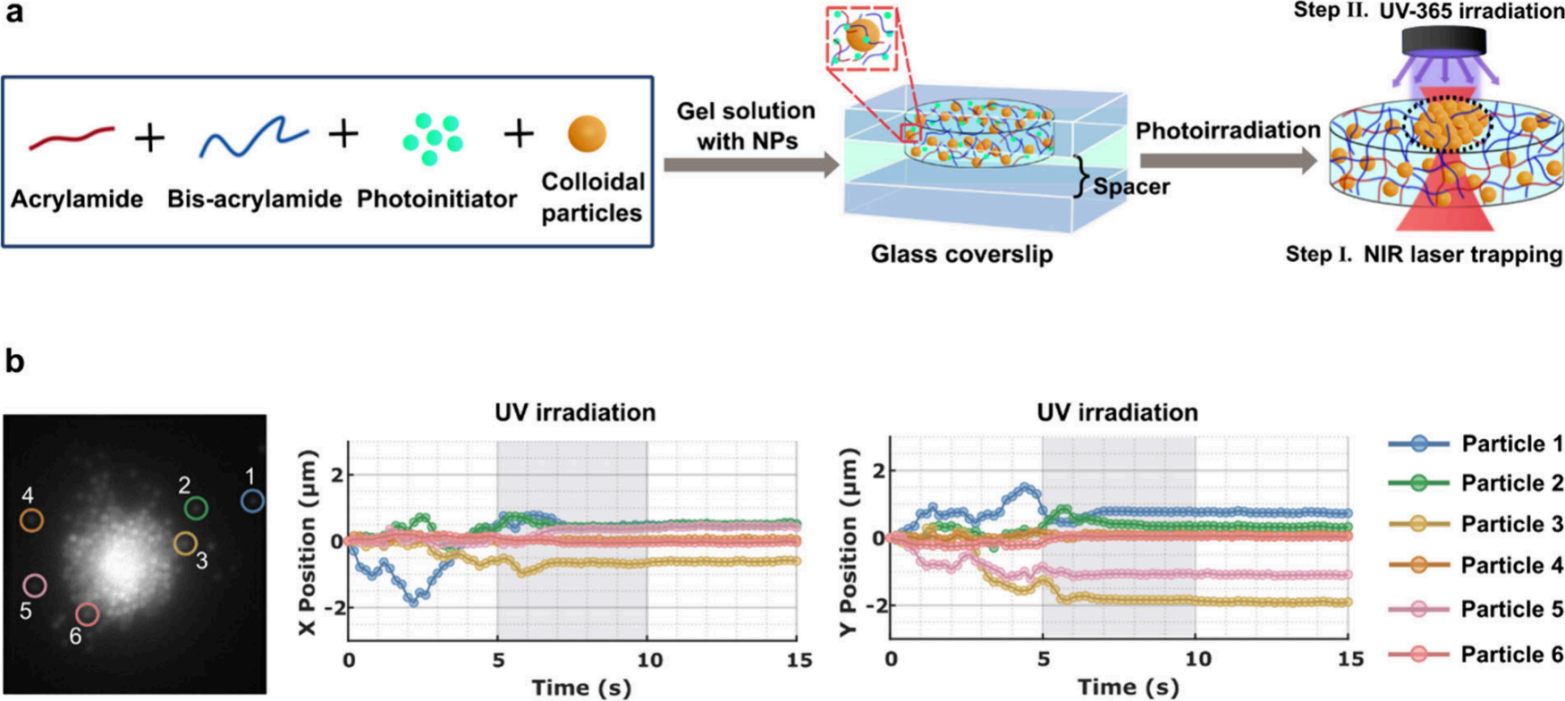
FRAME step 1 - Fixing of nonequilibrium optical matter in a photo-cross-linked hydrogel. (a) Method illustration: PPM solution containing 1 μm PSMPs is placed between two glass coverslips with a spacer, followed by simultaneous irradiation with an NIR trapping laser. Once the optical matter is assembled, and an additional UV light is switched on to fix the optical matter in the hydrogel. (b) Concept validation: A plot showing the X and Y movement of six particles from the assembly before, during, and after UV irradiation, demonstrating that the particle dynamics are fixed in the hydrogel after UV irradiation, based on Single Particle Tracking (SPT) analysis. The positions of six individual particles are marked in six distinct colors corresponding to the plot.

Figure 1b shows the evolution of X and Y positions of a few particles over time, confirming that we could successfully fix the colloidal assembly. Before UV irradiation, particles inside the OM are dynamic but move relatively slowly, constrained by the surrounding particles. Conversely, particles outside (particle 1–6, Figure 1b inset) are much more dynamic. Upon UV-365 light irradiation, photopolymerization occurs within 2 s, as indicated by the particle traces, which show a gradual decrease in fluctuations (Figure 1b). After UV irradiation, no movement is observed, confirming the fixation of the OM by photopolymerization (see Supporting Information, Movie S1). Images of the evolution of the assembly during the process are shown in Figure S3. Since the OM is fixed, it can be transported to other characterization tools (e.g., confocal, SEM, or other). We note here that while the method was showcased on OM, it can be applied to any other out-of-equilibrium colloidal self-assemblies, provided that they can be formed in the hydrogel solution.

### 2.2 Imaging of Fixed Colloidal Assemblies

FRAME’s second step is the high-resolution characterization of the fixed colloidal self-assembly. Here, we demonstrate it using 3D confocal microscopy and scanning electron microscopy (SEM), but other modalities may also be applicable. Since FRAME includes photopolymerization of the whole sample and postprocessing steps (necessary for SEM), it is crucial to ensure that these (post)-processes do not alter the assembled structure. To address this, we employed 1 μm fluorescent carboxylated PSMPs. Their size is significantly larger than the diffraction limit of optical microscopy, allowing for localization and comparison across different imaging modalities before and after FRAME processing.

Hence, we first recorded widefield microscopy images before and after gelation, confirming the absence of structural changes (Figure S3). Subsequently, we transferred the sample to a 3D confocal microscope and acquired a 3D image stack, successfully resolving the structural properties of the OM in three dimensions (Figure 2c,d). Finally, the same sample was imaged via SEM. Of note, the SEM imaging process is complex, involving, drying, sputtering with Au/Pd, and high vacuum conditions, all of which can induce structural changes in the sample.^63^ Hence, we overlaid the 3D confocal (preprocessing) and SEM (postprocessing), localized, and quantified the displacement of particles in the OM, finding a mean displacement error of 43 and 33 nm in the X and Y directions, respectively (Figure S4.1). For 3D correlation, we first acquired a confocal Z-stack before drying and then a second Z-stack after drying, following SEM preparation protocol, which included Au/Pd sputtering. We matched centroids (n = 121) using sphere-gated iterative closest point (ICP) procedure, and obtained localization precisions of approximately σ_X,Y_ ≈ 40 nm and σ_Z_ ≈ 87 nm, with no systematic bias (Figure S4.2). SEM provided high-resolution surface morphology and an independent XY centroid cross-check after high-vacuum imaging; volumetric (XYZ) registration and displacement metrics were derived from the paired confocal stacks. Together, these results confirm that the FRAME process does not measurably alter the assembly structure, with residuals consistent with centroid localization precision.

**Figure 2:**
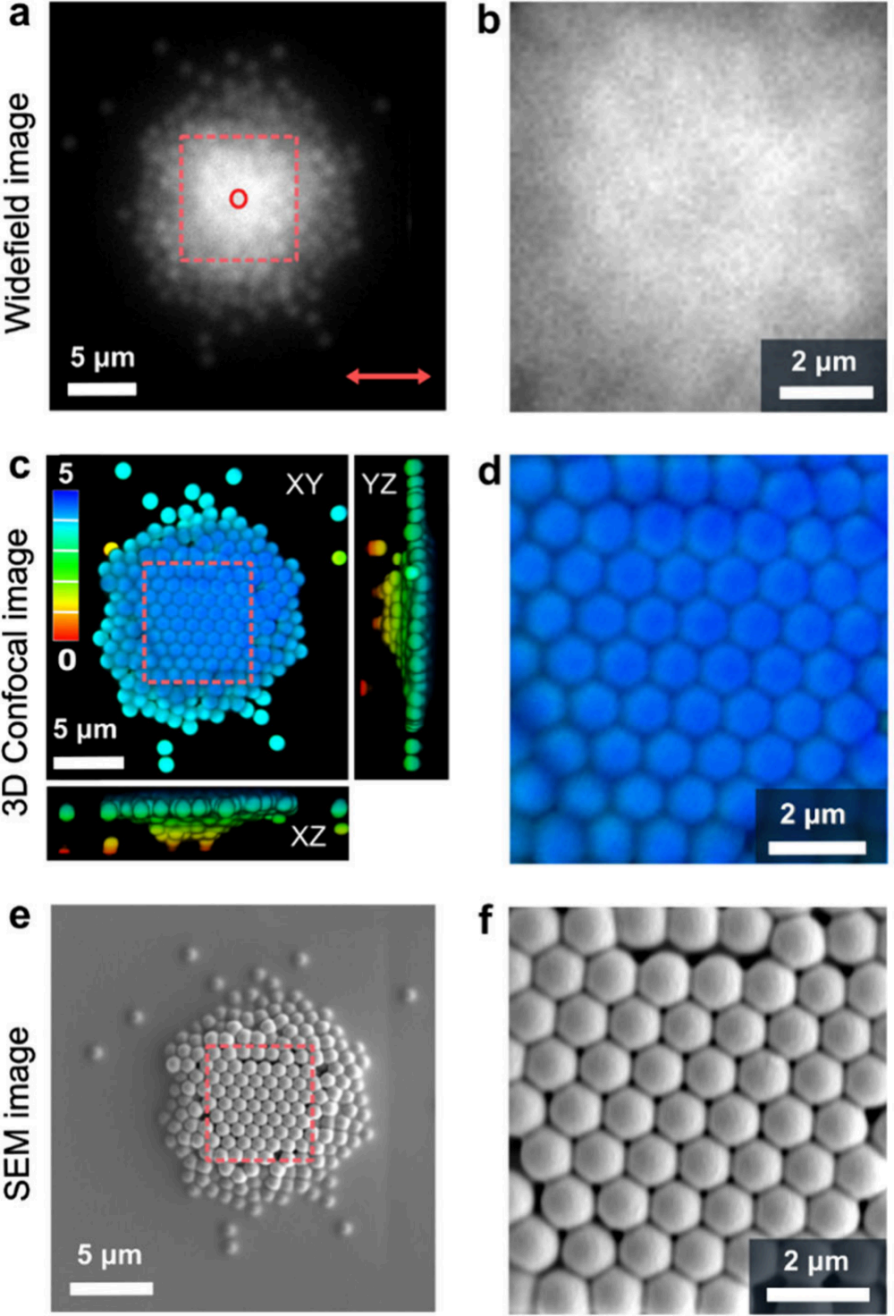
Method validation on fluorescent PSMPs (1 μm diameter). (a) Widefield microscopy image showing optical matter composed of 1 μm PSMPs, locked after photopolymerization. The red arrow indicates the polarization direction of the 1064 nm NIR trapping laser, and the red circle marks the NIR laser irradiation spot at the center. (c, e) 3D confocal and scanning electron microscopy (SEM) images illustrate the embedding of permanent optical matter with PSMPs in the polymer network. (b, d, f) Magnified views of regions from (a, c, e), respectively. Scale bars in (a, c, e) are 5 μm, and in (b, d, f) are 2 μm.

As demonstrated here, the FRAME methodology is robust enough to allow characterizing samples using various techniques in a correlative way, in this case 3D confocal microscopy and high-resolution SEM. We note here than other advanced techniques for structural or spectroscopic characterization (if applicable) may as well be used.

### 2.3 3D Structural Analysis

After validation of FRAME, we can now use it to characterize the chosen nonequilibrium OM system. The 3D confocal microscopy image provides detailed information on the three-dimensional structure of the assemblies through image processing. Briefly, the particles are localized using 3D Gaussian fitting. Then, the center-to-center distances between all possible particle pairs are calculated using a Euclidean distance matrix. Subsequently, each layer is analyzed iteratively to detect triangles that meet specific geometric criteria of common crystal structures (e.g., equilateral triangles with angles close to 60 degrees for hexagonal packing). These triangles are further analyzed across layers to identify 3D structures, including hexagonal prisms. This analysis enables the extraction of the “crystal” unit lattice and the packing density. In addition, it determines the degree of crystallinity of the structure by calculating the ratio of particles that belong to such crystal unit cells. In the case of structural analysis for the assembly of PSMPs (1 μm), a positional tolerance of ± 200 nm was applied. Particles located within this range above or below a reference plane were considered part of the same layer. Each layer contains both grayscale and colored particles, where the colored particles specifically contribute to the identified packing structure.

Figure 3 shows an example of OM structure formed, fixed by FRAME and then imaged in 3D using a confocal microscope (Figure 3a). Figure 3b shows a graphical representation of the 3D assembly, realized from the 3D localization positions and the known size of the particles. Only the particles that were found to be part of a crystalline structure are colored, representing approximately 80% of the entire assembly. The lattice arrangement follows an ABA packing pattern, with a decrease in particle number from the top surface to the bottom of the assembly. The c/a ratio refers to the ratio of the vertical distance between layers (c) to the horizontal distance between particles (a) in the hexagonal lattice, and it helps to describe how the lattice is stacked and spaced in three dimensions. The c/a ratio of the unit lattice is calculated at 1.71 ± 0.15 (Figure 3c), which is slightly higher than the theoretical lattice constant (1.63) for hexagonal close packing (HCP) structures. However, the packing efficiency is found to be approximately 71.6%, calculated by averaging the c/a values for the six sides, which is slightly lower than the ideal packing density (74%) of an HCP ABA structure (Figure 3c). The packing efficiency is calculated on the basis of unit cells in the central region of the assembly and therefore reflects local packing rather than a global packing fraction for the entire assembly.

**Figure 3:**
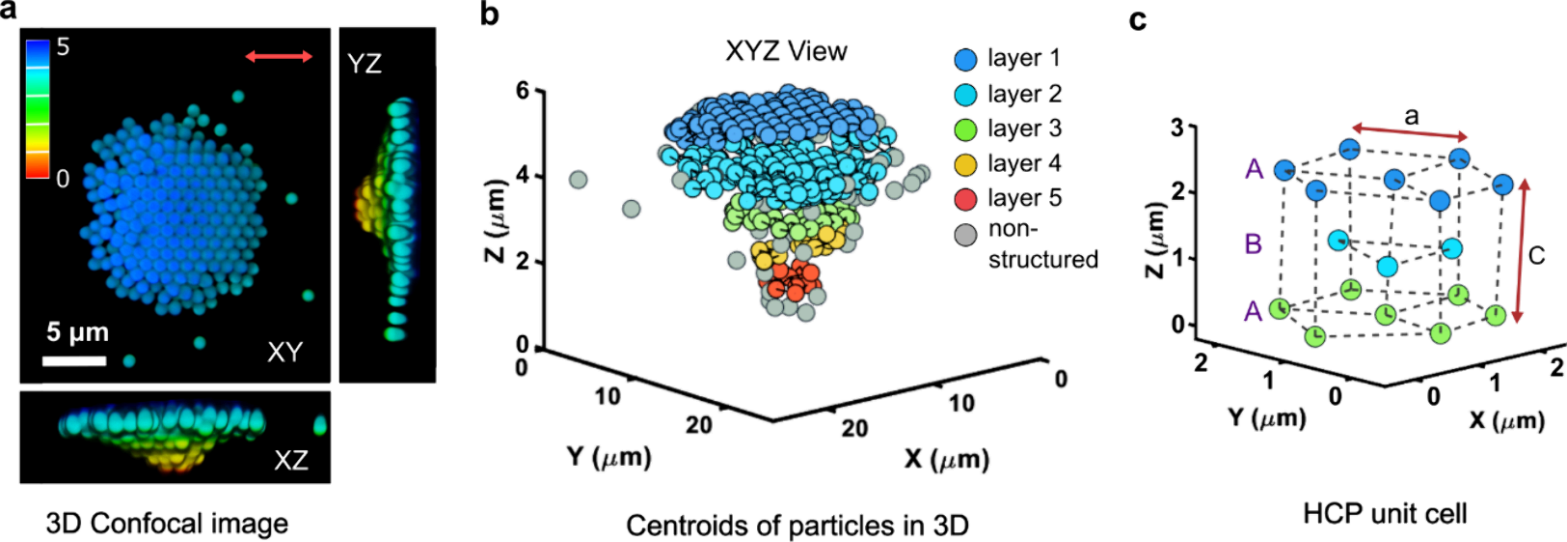
3D structural analysis of the permanent optical matter. (a) A 3D confocal microscopy image of the permanent optical matter formed by 1 μm PSMPs fixed in a hydrogel. The color bar in the confocal microscopy images indicates the Z-depth arrangement of the particles from top to bottom. (b) Euclidean distance matrix-based analysis used to extract individual layers of particle centroids, revealing specific structural arrangements. The color bar represents the number of particle layers in the assembly from top to bottom. Gray particles in the permanent optical matter are those not contributing to specific structural arrangements. (c) Hexagonal ABA unit cell extracted from the layer structures, showing the lattice parameters and individual particle centroids represented for the unit cell.

### 2.4 Controlling Particles’ Arrangement in Optical Matter Structures

Now that we can extract the 3D structure of nonequilibrium OM, this feedback can be used to better control the spatial arrangement of colloidal particles. This emphasizes the role of our method in characterizing and designing nonequilibrium colloidal assemblies. Crystallinity is a critical aspect of structural arrangement and refers to the degree of periodicity or order in the spatial organization of particles. Similarly, in OM particles organize themselves into specific structural arrangements, which can also be described in terms of crystallinity. In this study, we analyzed the local arrangement of particles within layers or regions of the assembly to identify specific crystalline patterns, including HCP and body-centered cubic (BCC) unit cells.

As mentioned earlier, OM structural arrangement for a specific particle type and size, is mostly dictated by the properties of the incident optical field (wavelength, polarization, power density, area irradiated···). Hence, we expect that the position of the trapping laser relative to the interface directly impacts the arrangement and crystallinity of the OM formed. FRAME provides an ideal technique to investigate this effect. By focusing the laser at different Z-depths (−1.5 μm, −2.5 μm, and −3.5 μm below the glass interface), we studied how the relative position of the laser focus to the interface influenced the 3D structure of the OM. The extraction of unit cells with the volumetric representation of each unit cell at different Z-depths is shown in Figure S5. From the backscattered intensity profile and simulations of the beam profile, we determined the beam waist diameter (*w*_0_) corresponding to a given power density (*I*_0_) (Figure S6).

At a Z-depth of −1.5 μm, corresponding to a power density of 14 MW/cm^2^, approximately 70% of the cases (7 out of 10 independent experiments) exhibited a concentric ring-like structure, while many particles did not contribute to any specific pattern in the OM (Figure 4a). In about 30% of the experiments (3 out of 10), a BCC structure was observed at the center of the OM (Figure 4b). At a Z-depth of −2.5 μm, HCP structures began to form at the center of the OM, although fewer particles contributed to the hexagonal arrangement (Figure 4c). Finally, at a Z-depth of −3.5 μm, the OM exhibited a well-ordered HCP structure with an ABA pattern (Figure 4d).

**Figure 4:**
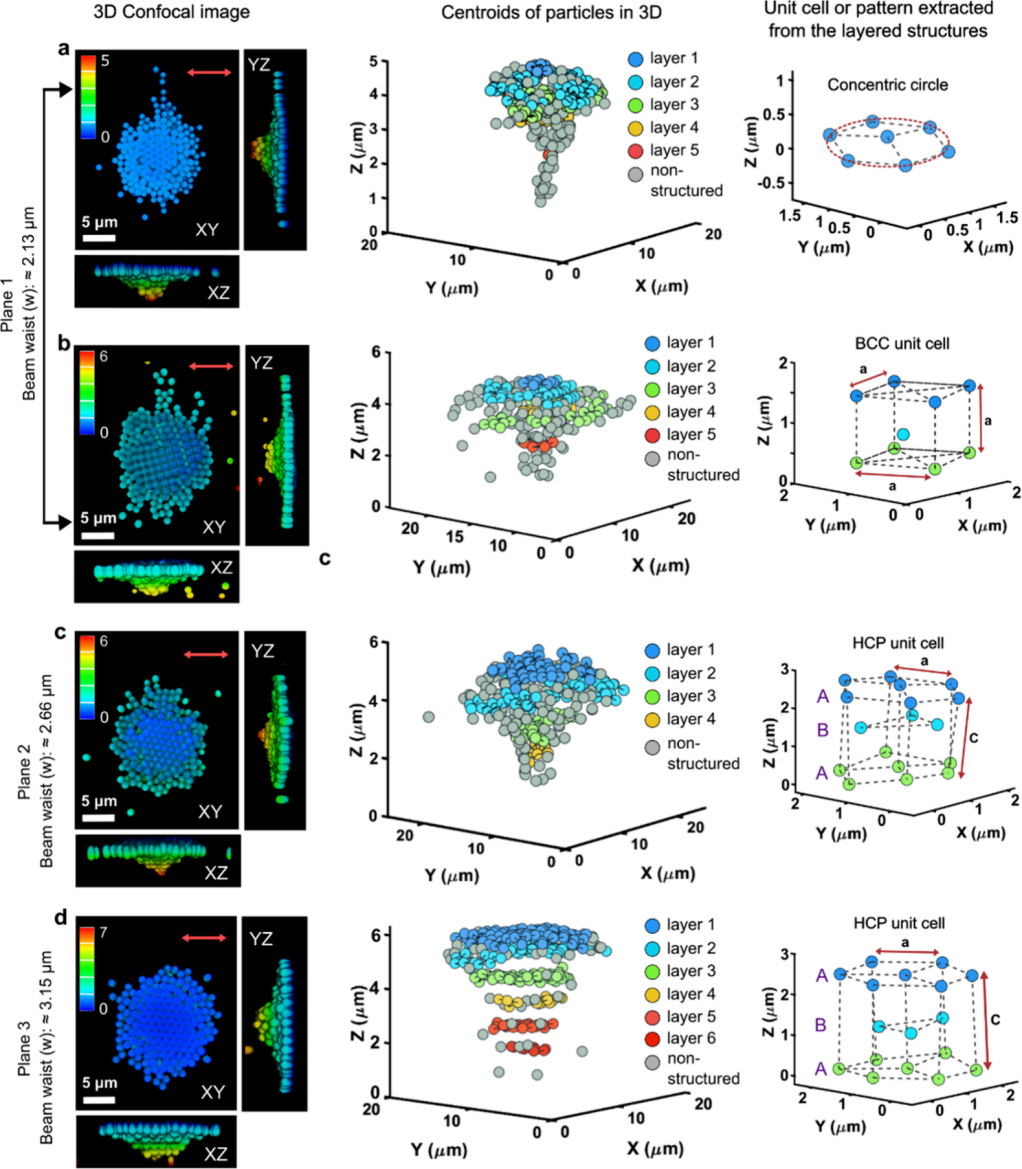
Locking dynamic optical matter formed at different Z-depths. (a, b) 3D confocal microscopy images showing a concentric circle structure, and a BCC (body-centered cubic) lattice unit cell pattern extracted from the layered structures of permanent optical matter, with a beam waist diameter of ∼2.13 μm. (c) 3D confocal microscopy image showing an HCP (hexagonal close-packed) ABA lattice obtained at an optical trap with a beam waist diameter of ∼2.66 μm. (d) 3D confocal microscopy image showing another HCP ABA lattice obtained at an optical trap with a beam waist diameter of ∼3.15 μm. The scale bars for all confocal microscopy images are 5 μm.

The analysis of the structures formed is detailed in Table 1, which summarizes the packing efficiencies (see Materials and Methods details) and c/a ratios for the different arrangements. The packing efficiency for the BCC structure observed at −1.5 μm was calculated to be 67%, which is very close to the theoretical packing efficiency of BCC of 68%.^64^ In contrast, the HCP structures formed at −2.5 μm had a packing efficiency of 63% and a c/a ratio of 1.739 ± 0.045, which is lower than the theoretical maximum packing efficiency of approximately 74% with a c/a ratio of 1.63. At −3.5 μm, the packing efficiency for the HCP arrangement was significantly higher at 70%, however, this value still deviated from theoretical expectations. We note here that since we are dealing with microparticles assembling into crystalline structures in a dynamic environment, it is not surprising that the packing efficiency is lower than the theoretical values expected for solid crystals formed by atoms bound through strong chemical bonds. In conventional materials, the high packing densities arise from the strength and directionality of atomic-scale electronic interactions (chemical bonds), which stabilize compact lattice structures. However, even in these systems, the packing efficiency can deviate from idealized theoretical approximations. For example, HCP metals such as titanium exhibit a c/a ratio of approximately 1.587, which is lower than the ideal value of 1.633. This deviation is attributed to directional bonding effects involving hybridized d-electron orbitals, which induce slight distortions from the ideal close-packed geometry.^65^

**Table 1:** Structural Parameters and Power Density Measurements of Optical Matter (OM) Formed at Various Z Depths^a^

Z-depth (μm)	Power density (MW/cm^2^)	Unit cell	*a*-axis Length (μm)	*c*-axis Length (μm)	c/a ratio	Packing efficiency (%)	Coordination number
–1.5	14.0	BCC	1.11 ± 0.01	N/A	N/A	66.7	8
–2.5	8.9	HCP	1.07 ± 0.03	2.04 ± 0.06	1.91 ± 0.07	63.2	12
–3.5	6.4	HCP	1.23 ± 0.03	2.13 ± 0.04	1.74 ± 0.05	69.7	12

a Abbreviations: Not Applicable (N/A), Body-Centered Cubic (BCC), and Hexagonal Close Packing (HCP).

Similarly, in OM the observed variations highlight the influence of trapping conditions and the properties of the light used on the structural arrangement of the OM. Yet, even under favorable conditions, small deviations from ideal crystalline order can arise due to thermal fluctuations, the relatively weak nature of optical binding forces, and localized scattering-induced shifts. While these factors do not prevent the formation of assemblies, they can reduce the degree of structural order, resulting in packing efficiencies slightly below those seen in ideal atomic systems. Moreover, the number of scattered photons per unit area gradually decreases, moving away from the focus, resulting in weaker and less ordered structure at the periphery of the assembly, also coherent with the observed structure.

### 2.5 Fixing Dynamic Optical Matter with Subdiffraction Limit Nanoparticles

One of the factors limiting the characterization of nonequilibrium colloidal assemblies, including OM structures, is not only their dynamics but also their relatively high packing density. This becomes problematic for standard optical microscopy methods when the particle size approaches or falls below the diffraction limit of light (approximately 200–300 nm). Resolving individual particles in a densely packed, dynamic assembly becomes particularly challenging under these conditions, as the diffraction limit makes signals overlap. FRAME is also useful for this condition. To demonstrate this, we used carboxylate-coated fluorescent polystyrene nanoparticles (PSNPs) with diameters of 300 and 200 nm. After 20 min of NIR laser irradiation, a large-scale assembly structure was formed and locked using FRAME. Subsequently, we employed 3D confocal microscopy and advanced SEM imaging to resolve the structure of the resulting permanent OM.

As expected, the structure could not be resolved using 3D confocal microscopy, probably due to the aforementioned size and density limitations (Figure S7). However, SEM imaging of the OM formed by 300 nm PSNPs revealed a highly packed structure with tightly arranged particles, featuring small structural subunits distributed across the assembly (Figure 5a). For the 200 nm PSNPs, a random 3D packing arrangement was observed, characterized by a dense, close-packed structure. This observation suggests that, despite the randomness in particle placement, the assembly achieves relatively high packing efficiency, as seen in the SEM images (Figure 5d). Since SEM only captures surface details, the internal structure of the assembly cannot be directly resolved. However, using the z-depth information from 3D confocal imaging, the overall packing height was determined to be approximately 2.2 and 2.0 μm for the 300 and 200 nm PSNPs, respectively (Figure S7).

**Figure 5:**
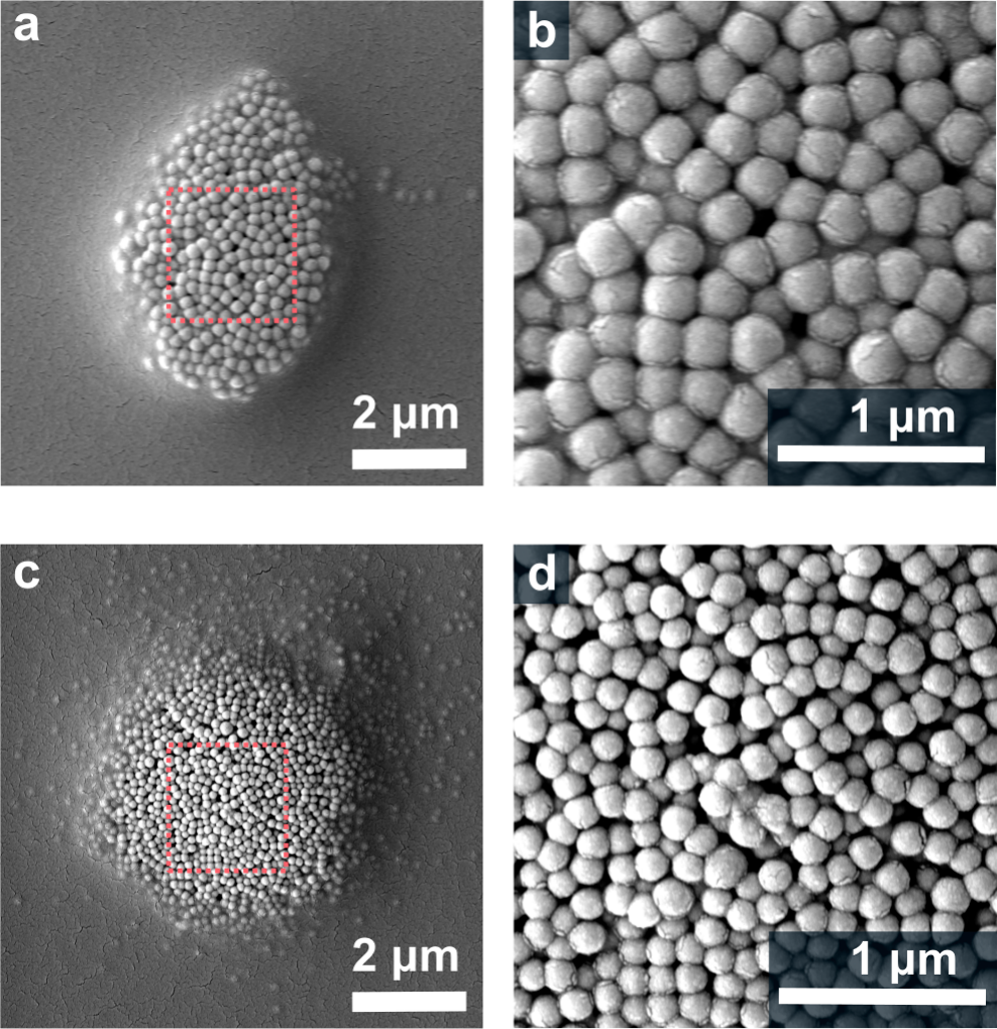
Locking dynamic optical matter composed of subdiffraction limit nanoparticles. (a, b) SEM images showing the assembly of 300 nm polystyrene (PS) nanoparticles within the polymer network, with (b) providing a magnified view of the structure. (c, d) SEM images displaying the assembly of 200 nm PSNPs within the polymer network, with (d) showing a closer view of the respective structure. The scale bar in (a, c) represents 2 μm, and in (b, d), it represents 1 μm.

### 2.6 Fixation and Super-Resolution Imaging of Optical Matter Formed by Subdiffraction Limit Nanoparticles

For assemblies composed of subdiffraction-sized nanoparticles, conventional confocal microscopy cannot resolve individual particles and their internal ordering in 3D, while SEM only reveals the outermost layer of the assembly. To overcome these limitations, we performed 3D STED imaging on FRAME-fixed assemblies of 200 nm polystyrene fluorescent nanoparticles (Figure 6). Deconvolved 3D STED Z-stacks clearly resolve individual particles throughout the whole assembly, in contrast to the blurred confocal projection of the same structure (Figure 6a,b), and the orthogonal XZ/YZ views reveal the layered structure and its axial extent (Figure 6c). For quantitative analysis, the intensity distribution of each particle was fitted with a 3D Gaussian to obtain subpixel centroid coordinates, which were then used to construct depth-color-coded centroid projections in XY (Figure 6d) and in XZ/YZ (Figure 6e), and to calculate Euclidean nearest-neighbor (NN) distances. The resulting in-plane (XY) NN center-to-center distances show a distribution centered at ≈177 nm with a standard deviation of ≈86 nm, consistent with dense, multilayer packing with only short-range order (see Supporting Information, Section S8). This reduced mean and broad spread arise because the assembly is 3D and multilayered, so nearest neighbors are not confined to a single plane and the XY-projected separations can be smaller than the nominal particle diameter. At the same time, the corresponding Fourier analysis exhibits a diffuse ring rather than sharp Bragg peaks, indicative of short-range order and the absence of pronounced long-range crystallinity (see Supporting Information, Figure S8d). Together, these measurements provide a quantitative description of the internal three-dimensional ordering in the nanoparticle assembly.

**Figure 6:**
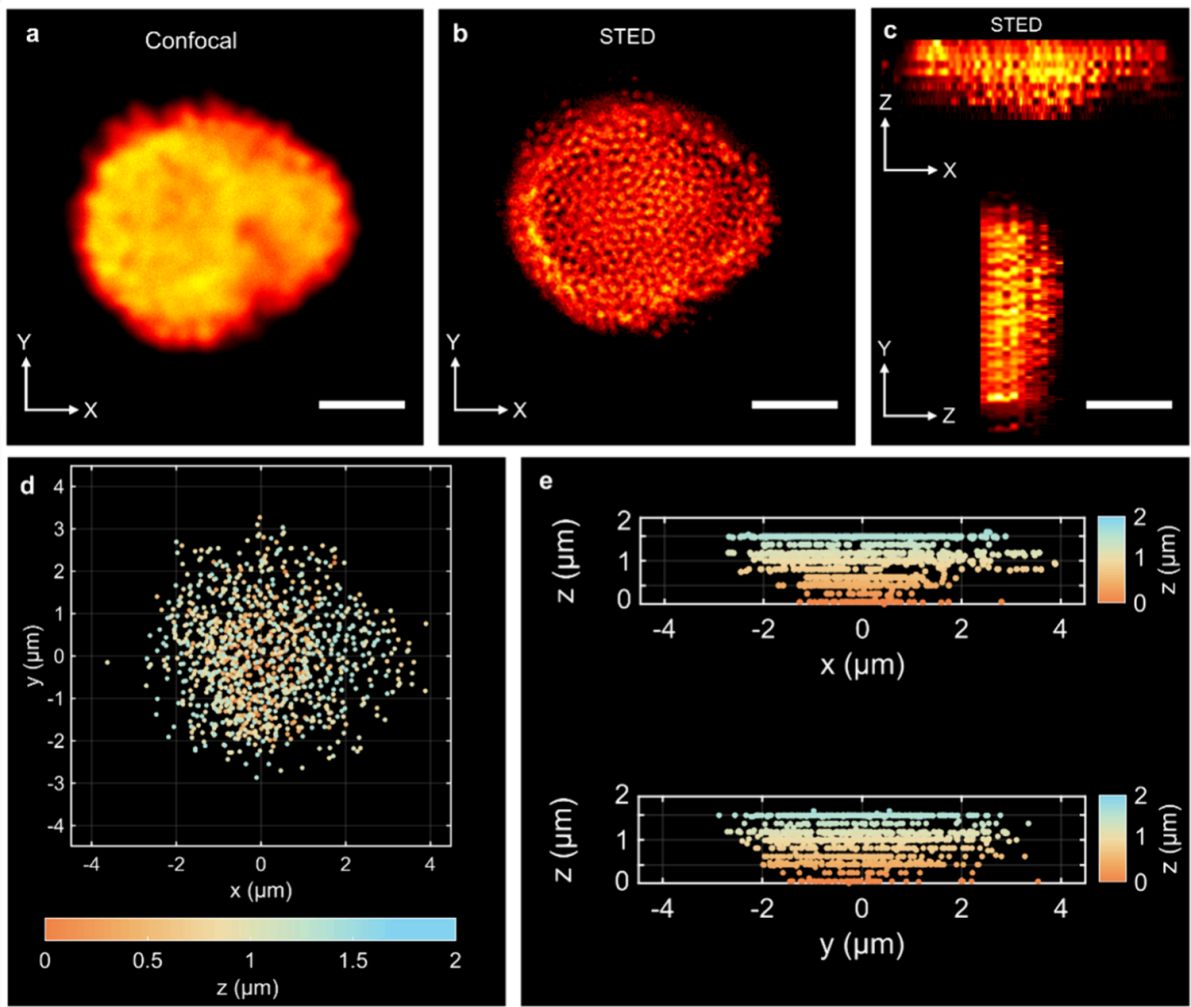
Confocal and STED imaging of a FRAME-fixed assembly of 200 nm fluorescent nanoparticles. (a) Confocal XY image of the FRAME-fixed assembly, where the assembly appears as a blurred aggregate and individual particles cannot be resolved. (b) Deconvolved STED XY image of the same assembly, resolving individual 200 nm particles and their dense packing. (c) Orthogonal XZ and YZ views from the STED Z-stack, revealing the three-dimensional extent layer structure of the nanoparticle assembly. Each scale bar represents 2 μm. (d) XY projection of STED-localized particle centroids; each point marks one centroid and is color-coded by axial position z (μm), illustrating depth-dependent layering. (e) XZ (top) and YZ (bottom) projections of the STED-localized centroids, with each point color-coded by axial position z (μm), highlighting the vertical layering and lateral spreading of the nanoparticle assembly.

## 3 Discussion

FRAME, with its rapid photopolymerization (less than 2 s) and fine mesh size effectively preserves the structural integrity of nonequilibrium colloidal assemblies. It is important to acknowledge that, given the inherently nonequilibrium nature of these systems, the structure captured by FRAME represents a snapshot of a specific arrangement of the assembly. To fully understand the range of configurations occurring dynamically, multiple samples need to be immobilized and analyzed according to the ergodicity hypothesis.^66^ Despite this, FRAME significantly simplifies the characterization of nonequilibrium self-assemblies by decoupling the trapping from the analysis using high-resolution imaging techniques. We note here that spectroscopic methods (if applicable) could also be performed on fixed colloidal assemblies after FRAME without the need for complex in situ trapping accessories. Because the assemblies are immobilized by FRAME, they can be transferred to dedicated dark-field spectroscopy setups optimized for NIR detection, enabling measurements over a broader spectral range and a more detailed mapping of their optical response. In this study, we report dark-field scattering spectra as a first step; a full NIR-optimized characterization can be pursued in future work (see Section S9). Hence, while our focus has been on imaging and structural analysis, FRAME is versatile and can be integrated with a variety of other characterization techniques. Likewise, FRAME is applicable to particles composed of different material systems, including dielectric (polystyrene, silica) and metallic (gold) particles. Figure S10 presents assemblies of 1 μm silica microparticles and 400 nm gold nanoparticles embedded in a PAA hydrogel and fixed by FRAME, demonstrating the method’s applicability across materials. FRAME also works robustly across different trapping wavelengths and polarizations, as 1 μm PSMPs assemblies formed with 780 nm linear and 1064 nm circular traps are both successfully fixed and imaged (Section S15). Furthermore, FRAME facilitates optical and spectroscopic measurements such as refractive index mapping and absorption studies on stabilized colloidal assemblies without the need for complex in situ trapping setups. This is particularly advantageous for studying colloidal metamaterials (explained in the introduction), where understanding and tuning light–matter interactions is crucial. In addition, FRAME-fixed assemblies also remain stable under ambient storage; reimaging after 528 days showed no measurable structural drift (Figure S11).

FRAME enables high-resolution structural characterization, providing crucial feedback for understanding the structural arrangements of colloidal self-assemblies (in particular OM), and rationally designing materials for specific applications. Using this approach, we discovered that the unit cell and crystallinity of nonequilibrium OM structures can be manipulated by adjusting the laser focus position relative to the interface. This adjustment alters the irradiation area and power density at the interface, resulting in concentric circle, BCC, or HCP crystal structures. These insights could only be obtained with FRAME, making it a key tool for the rational design of nonequilibrium assemblies.

Moreover, our results provide the first detailed structural insight into small nanoparticle assemblies. Although previous studies indicated the formation of such assemblies,^28,29,67^ the exact structural details were unclear due to the limitations of optical microscopy and the inability to apply other characterization methods as the formation and the structural characterization of OM had to be simultaneously performed. With a mesh size smaller than 23 nm, FRAME can accommodate subdiffraction-limit sized-particles without diffusion. Indeed, FRAME has revealed that smaller nanoparticles exhibit more chaotic arrangements, lacking clear crystalline patterns. This can be attributed to two factors: smaller particles have higher diffusion coefficients, leading to increased entropy and a greater energy requirement to maintain order. Furthermore, the light-matter interactions driving the assembly process, including multiple scattering, scale with particle volume and thus these forces are significantly weaker in smaller particles. This challenge has led recent OM studies to focus on metallic particles, which have much stronger light-matter interactions due to their plasmonic properties, that enhances their scattering cross-section by more than 1 order of magnitude.^45,68,69^ This further demonstrates the robustness of the FRAME method, which enables structural analysis of OM assemblies ranging from 1 μm microparticles down to subdiffraction-limit nanoparticles as small as 200 nm. Moreover, given that the physicochemical properties of the gel solution are similar to neat water in terms of transparency and viscosity, it is reasonable to envision that fixing nonequilibrium assemblies formed in the presence of other fields such as magnetic, electric or chemical fields, or other strategies including acoustic manipulation, thermophoretic assembly, diffusiophoretic migration, and flow-induced organization, would also be possible through the FRAME method.

Nonequilibrium colloidal self-assemblies are particularly intriguing because, unlike their equilibrium counterparts, they can be dynamically tuned, reconfigured, and exhibit unique collective behaviors including flocking, swarming, or propagating waves.^2,70–72^ These behaviors involve interactions between colloidal particles and with their surrounding medium, making them ideal, controllable model systems to investigate and understand complex multibody system such as swarming nanorobots.^20^ FRAME allows for high-resolution characterization of these transient structures providing feedback for rational design of these complex colloidal systems. This fixation capability allows for the use of a wide range of advanced characterization techniques that are typically not compatible with dynamic or liquid-phase systems. For example, once the assemblies are fixed, high-resolution SEM, X-ray scattering, or even super-resolution fluorescence microscopy (with appropriate labeling) can be employed to investigate their internal structure in detail. This opens up opportunities for structural analyses that were previously inaccessible for nonequilibrium assemblies. Moreover, such nonequilibrium colloidal assemblies are of particular interest due to their dynamic and reconfigurable nature, and FRAME enables their high-resolution capture, supporting the design of complex active matter systems.

## 4 Conclusion

We demonstrated FRAME (Fixation and Resolving of Colloidal Active Matter Ensembles), an adaptable method for fixing and characterizing nonequilibrium colloidal self-assembly via fast photopolymerization. The rate of photopolymerization and the small mesh size ensured that the structure stayed intact as validated by comparing different image modality (optical microscopy, SEM) at different stage of the workflow. FRAME was validated on OM, a nonequilibrium colloidal self-assembly assembled by optical binding. We showed that FRAME enables the decoupling of the nonequilibrium self-assembly formation (here, OM) and its characterization, which opens the door to the use of a plethora of high-resolution characterization methods. Thus, FRAME provides crucial feedback for controlling structural arrangements and rationally designing materials for specific applications. This was demonstrated by forming nonequilibrium OM assembly using different beam waists which resulted in different crystalline structures ranging from concentric circle to hexagonal packing. Thanks to FRAME, we determined that placing the beam focus deeper inside the glass, increasing the beam waist at the interface, can promote better packing efficiency of OM assembly. Finally, we were able to observe the detailed structure of OM structure formed by particles smaller than the diffraction limit demonstrating that their high entropy and weakened light matter interaction made them unable to form an ordered crystalline structure. FRAME enabled crucial insight that would not have been possible by studying these assemblies on their dynamic form. While FRAME allows for high-resolution characterization of these transient structures, we note that it can also be integrated with other imaging techniques or even spectroscopic methods. Moreover, by fixing the self-assembly, FRAME provides the opportunity to stabilize arrangements that are not thermodynamically favorable, also giving the possibility to obtain a nonequilibrium in absence of external stimuli, for example, the structure of the OM was stabilized even without the optical field that created it. These capabilities position FRAME as a powerful tool for the development of self-assembled colloidal crystals and optical metamaterials with tunable optical properties for advanced photonic applications. FRAME’s possibility for characterization, feedback for rational design and stabilization of nonequilibrium structure without their stimuli makes it a useful tool in colloidal science. These results suggest that FRAME can facilitate understanding of nonequilibrium assembly and support rational design for targeted applications.

## 5 Materials and Methods

### 5.1 Materials

The carboxylate-coated polystyrene latex beads were purchased from Thermo Fisher Scientific (Carboxylate-Labeled Microspheres, 0.2, 0.3, and 1 μm, yellow-green fluorescent 505/515, 1% solids). For 3D STED imaging, 200 nm fluorescent PSNPs were purchased as Invitrogen FluoSpheres Carboxylate-Modified Microspheres (Crimson, 625/645 nm; Thermo Fisher Scientific). The PPM solution was prepared using 12.5 wt % acrylamide (≥99%, Sigma-Aldrich, Germany), 0.3 wt % N,N’-methylenebis­(acrylamide) (≥99.5%, Sigma-Aldrich, Germany), 0.44 wt % lithium phenyl-2,4,6-trimethylbenzoylphosphinate photoinitiator (≥95%, Sigma-Aldrich, Germany), and Milli-Q water.

### 5.2 Optical Trapping Configuration

The optical trapping experiments were conducted using a conventional widefield setup with an optical trap. A 488 nm laser (100 mW, Spectra-Physics) served as the excitation source, focused through a widefield lens onto the back aperture of an objective lens (NA = 0.90, Olympus UPLFLN 60X Objective), with images obtained using an sCMOS camera. For trapping, a 1064 nm NIR laser (Laser Quantum Opus 1064, UK) was focused at the same back aperture via a beam expander and mirrors ().

### 5.3 Sample Preparation, Photopolymerization, and Postprocessing

The coverslip was cleaned using UV-ozone treatment for 60 min to ensure a clean glass surface. The top coverslip was made hydrophobic using Sigmacote (a siliconizing reagent), purchased from Sigma-Aldrich, Germany. The Polyacrylamide Photocuring Medium (PPM) solution containing nanoparticles was placed between glass coverslips using imaging spacers. Double-sided imaging spacers with a thickness of 0.12 mm and a well diameter of 20 mm (Grace Bio-Laboratories SecureSeal imaging spacer) were used for sample preparation. To achieve fast photopolymerization, the sample was irradiated with UV-365 light (M365L3-C1–365 nm collimated LED, Thorlabs) from a focused LED at a power density of 25 mW/cm^2^. After polymerization, the top coverslip was carefully removed from the hydrogel due to its hydrophobic surface. 3D confocal imaging was subsequently performed as described below. For scanning electron microscopy (SEM) imaging, the sample was air-dried for 3 h and then sputtered with a thin layer of Au/Pd for 60 s using a JEOL JFC-1300 Automatic Sputter Coater.

### 5.4 Optical and Electron Microscopy

The 3D confocal microscopy measurements were performed using a Leica SP8 confocal microscopy setup (TCS SP8 multiphoton system). 3D Z-stack imaging was conducted with a 100x oil objective (HC PL APO 100*x*/1.40–0.70 OIL) and hybrid detectors. A white light laser was used as the excitation source, with an excitation wavelength of 488 nm. The Z-stack scan was performed with a line averaging of 3, an image acquisition speed of 100, and a Z-step size of 100 nm. SPT experiments were carried out using a widefield multiplane microscopy setup capable of scanning a Z-depth of 5 μm. A schematic of the optical setup is illustrated in Figure S14. 3D STED imaging was performed on a Leica TCS SP8X microscope equipped with a HC PL APO 100×/1.40 oil-immersion objective (Leica Microsystems GmbH). The PSNPs were excited with a supercontinuum white light laser (NKT Photonics) at 633 nm wavelength with 80 MHz repetition rate. For emission depletion a pulsed 775 nm laser (Onefive, 80 MHz repetition rate), operated at 80% of its nominal power, was used to achieve super-resolved lateral (XY) resolution with a doughnut-shaped depletion pattern. Emission was recorded using a hybrid detector (HyD SMD, Leica Microsystems GmbH), with 16-frame averaging and a scan speed of 100 Hz for each optical section of the Z-stack. Images were sampled at 12 nm per pixel in XY with a 150 nm Z-step and acquired using Leica Application Suite X (LAS X). Electron microscopy experiments were conducted using a JSM-7200F field emission scanning electron microscope. The refractive index of the PPM solution was measured with a refractometer equipped with the same UV-365 LED.

### 5.5 Quantitative Structural Validation of FRAME-Fixed Assemblies

#### 5.5.1 Confocal/SEM Correlation

Segmentation analysis was performed on the SEM images (Figure S4.1), and the centroids (*Cx*, *Cy*) were mathematically calculated based on the center of mass of the pixels constituting the particles (see Section S4.2, Supporting Information). The centroid of the confocal microscopy image was determined by 2D Gaussian fittings. The centroids from the confocal and SEM images were overlaid to calculate the particle shift observed in SEM relative to confocal imaging.

#### 5.5.2 3D Correlation of FRAME-Fixed Assemblies before and after Drying

To assess possible *Z*-axis distortions and anisotropic shrink/swell during drying, we acquired confocal Z-stacks of the same 1 μm PSMPs assembly in hydrated PAA hydrogel and after drying with sputtering (prior to SEM). Stacks were rigidly registered by aligning XY projections (multimodal metric) and refining the axial offset via cross-correlation, then the transform was applied to the full volumes. Particle centroids were localized and matched one-to-one using a sphere-gated ICP (140 before/137 after; 121 matches). The deviations were σ_X,Y_ ≈ 40 nm and σ_Z_ ≈ 87 nm with error histograms centered at zero, indicating minimal, essentially isotropic change (<100 nm) upon drying.

### 5.6 Euclidian Based Structural Analysis

The centroid of each particle was obtained through 3D Gaussian fitting of the confocal microscopy images. The process began with the preparation of the particle position data, where the 3D coordinates (x, y, z) of the particles were loaded into a matrix. These data were initially in pixel units, so a conversion factor was applied to transform these values into physical units, including micrometers. Next, the particles were segmented into distinct layers based on their Z-coordinates (Figure 3b). This was achieved by defining a specific layer thickness, with particles within a certain Z-range grouped into the same layer. A Z-error margin of 20–30% was also considered to account for minor variations in the Z-coordinate, ensuring that particles within a specific range were correctly identified as part of the same layer.

Once the layers are defined, a Euclidean distance matrix is computed for each layer. This matrix contains the distances between every pair of particles within the layer, calculated using eq 1:

1 $$d_{ij}=\sqrt{{(x_{i}-x_{j})}^{2}+{(y_{i}-y_{j})}^{2}}$$

where *d*_*ij*_ is the distance between particles *i* and *j* and *x* and *y* are their respective coordinates. This calculation is crucial for identifying equilateral triangles formed by neighboring particles, which are indicative of hexagonal packing.

### 5.7 Calculation of Packing Efficiency

To calculate the packing efficiency, we compared the volume occupied by the particles to the volume of the unit cell, with the parameters obtained from particle localization.

For the BCC structure, the unit cell contains two particles: one at the center and $8\times \frac{1}{8}$ from the corners. From the distances between particles, the lattice parameter (*a*) is estimated. Using the known particle radius (*r*), the volume of a single particle is calculated. The total volume occupied by particles in the unit cell is $2\times \frac{4}{3}\pi r^{3}$ , and the unit cell volume is *a* ^3^. The packing efficiency is the ratio of the particle volume to the unit cell volume, multiplied by 100%.

For the HCP structure, the packing involves particles arranged in a hexagonal lattice, with six particles per unit cell. The lattice parameters *a* (lattice edge length) and *c* (cell height) are extracted from localization, providing the unit cell volume as $\frac{\surd 3}{2}a^{2}c$ . The atomic radius (*r*) related to the *a* by $r=\frac{a}{2}$ . The volume of single particle is $\frac{4}{3}\pi r^{3}$ , and the unit cell volume is $6\times \frac{4}{3}\pi r^{3}$ . The packing efficiency is calculated by dividing the total particle volume by the unit cell volume and multiplying by 100%.

### 5.8 Beam Waist Calibration

The trapping beam waist was calibrated using the same optical configuration as in our previous work (ref 27, section “Four nanoparticle system”). Briefly, the focal spot was imaged through the objective onto a camera and fitted with a Gaussian profile, yielding a 1/e^2^ beam waist diameter of ≈1.8 μm at focus. This calibrated waist value was then used as input for the Gaussian beam propagation analysis described in Supporting Information, Section S6.

## Supplementary Material





## Notes

Nanoparticle diffusion and localization-precision analyses before and after photopolymerization and fixation; control experiments for optically trapped assemblies in water and polyacrylamide photocuring medium (PPM); time-resolved optical-matter formation with polystyrene microparticles; structural validation of FRAME-fixed assemblies (confocal/SEM correlation and 3D comparisons before and after drying); 3D structural analyses of optical matter; beam-waist diameter measurements; 3D confocal imaging of assemblies containing subdiffraction nanoparticles; 3D STED super-resolution imaging of FRAME-fixed 200 nm nanoparticle assemblies; dark-field scattering spectra of FRAME-fixed microparticle and nanoparticle assemblies; FRAME fixation using silica and gold nanoparticles; long-term stability tests of FRAME-fixed assemblies under ambient conditions; descriptions of the widefield optical trapping and widefield-multiplane experimental setups; quantification of UV-365-induced radical formation in LAP; FRAME fixation under varied trapping wavelengths and polarizations (PDF)
Supplementary Movie S1 illustrating the fixation of PSMPs optical matter (AVI)
