# Supplementary material for: High Resolution Imaging of Nonequilibrium Colloidal Self-Assembly via Photofixation
Source: ACS Nano. 2026 Feb 12;20(7):6287–99. doi: 10.1021/acsnano.5c22002 (PMC12947731; doi:10.1021/acsnano.5c22002)
Supplement: Supplementary file 1 [file nn5c22002_si_001.pdf]

Supporting Information:

High resolution imaging of non-equilibrium colloidal self-assembly via photo-fixation

Jagannath Satpathy[a], Jim Jui-Kai Chen[a], Gang Wen[a],[b], Hiroshi Masuhara[c], Sudipta Seth[a], Volker Leen[e], Susana Rocha[a], Johan Hofkens[a],[f], Boris Louis*[a], Roger Bresolí-Obach*[a],[d]*

[a] Laboratory for Photochemistry and Spectroscopy, Division for Molecular Imaging and Photonics, Department of Chemistry, KU Leuven, Leuven 3001, Belgium.

[b] Department of Biotechnology and Biophysics, Biocenter, University of Würzburg, Am Hubland, 97074 Würzburg, Germany.

[c] Department of Applied Chemistry and Center for Emergent Functional Matter Science, National Yang Ming Chiao Tung University, Hsinchu 300093, Taiwan.

[d] AppLightChem, Department of Analytical and Applied Chemistry, Institut Químic de Sarrià, Universitat Ramon Llull, Via Augusta 390, Barcelona 08017, Spain.

[e] Chrometra Scientific B.V., Merelnest 3, 3470 Kortenaken, Belgium.

[f] Max Planck Institute for Polymer Research, 55128 Mainz, Germany.

Table of Contents

Section S1: Nanoparticle diffusion and localization precision studies 2

S1.1: 3D diffusion analysis of nanoparticles before and after polymerization 2

S1.2: Control experiment on localization precision after fixation 4

Section S2: Control experiment for optically trapped assemblies in water and PPM 7

Section S3: Time evolution of optical matter formation with polystyrene microparticles 8

Section S4: Quantitative Structural Validation of FRAME-Fixed Assemblies 9

S4.1: Confocal/SEM correlation 9

S4.2: 3D correlation of FRAME-fixed assemblies before and after drying 10

Section S5: 3D structural analysis of optical matter 12

Section S6: Measurement of beam waist diameter 13

Section S7: 3D confocal image of optical matter with sub-diffraction limit nanoparticles 14

Section S8: 3D STED super-resolution structural analysis of FRAME-fixed 200 nm PSNPs assemblies 15

Section S9: Dark-field scattering spectra of FRAME-fixed 1 µm PSMPs and 200 nm PSNPs assemblies ..17

Section S10: Fixation of optical matter with Silica and gold nanoparticles using FRAME 18
Section S11: Long-term stability of FRAME-fixed assemblies under ambient storage 19
Section S12: Widefield experimental setup with integrated optical trap 20
Section S13: Power-dependent radical formation in LAP under UV-365 irradiation 21
Section S14: Widefield-multiplane experimental setup 22
Section S15: FRAME fixation of PSMPs formed at different trapping wavelengths and polarizations 23
Section S16: Fixation of optical matter by FRAME (Supplementary Movie S1) 24

Section S1: Nanoparticle diffusion and localization precision studies

S1.1: 3D diffusion analysis of nanoparticles before and after polymerization

In this section, we discuss the rapid three-dimensional (3D) tracking and diffusion analysis of nanoparticles before and after polymerization. We use a custom-built, multiplane widefield microscopy setup (see Figure S14) that allows for tracking of nanoparticles within a depth of 5 µm in the solution. Fluorescent carboxylate-coated polystyrene nanoparticles (PSNPs), sized at 23, 200, and 500 nm (excitation/emission wavelengths of 488/515 nm), are used in these experiments. Employing a range of particle sizes aids in understanding the nanoparticle fixation phenomenon following photopolymerization. Sample preparation follows the protocol outlined in the Methods section. A 488 nm laser, with a power density of 0.1 mW/cm², is used as the excitation source. Upon widefield laser irradiation, fluorescence intensity from individual particles is observed, with localization in the Z-depth plane correlating to the maximum intensity emitted by each particle. Particle diffusion in solution is recorded for 5 seconds at an acquisition rate of 100 frames per second (fps). Subsequently, UV irradiation at 365 nm for 5 seconds triggers photopolymerization, and we record the process at 100 fps for 5 minutes. This extended observation post-polymerization is essential to ensure accurate particle fixation within the hydrogel matrix.

The diffusion of particles in a solution is determined by analyzing the time dependence of their mean squared displacement (MSD). The MSD is derived from the particles' mean free path in the solution, described by the following equation:

$$MSD(\tau) =< \Delta r(\tau)^2 >=< \left[\left(r(t+\tau) - r(t)\right)\right]^2 >$$

Where r stands for the position of the particle obtained from localization, τ is the delay time, and t is the starting time of the diffusing particle. The MSD can also be written as:

$$MSD(\tau) = 2nD_n\tau$$

Where n denotes the dimensionality of the diffusion process, D is the diffusion coefficient, and t represents the elapsed time. The process for localization of each particle in the different depth planes in the multiplane-system is reported in the previous study, where the 3D fast-tracking of the particle inside the solution with better precision is explained.

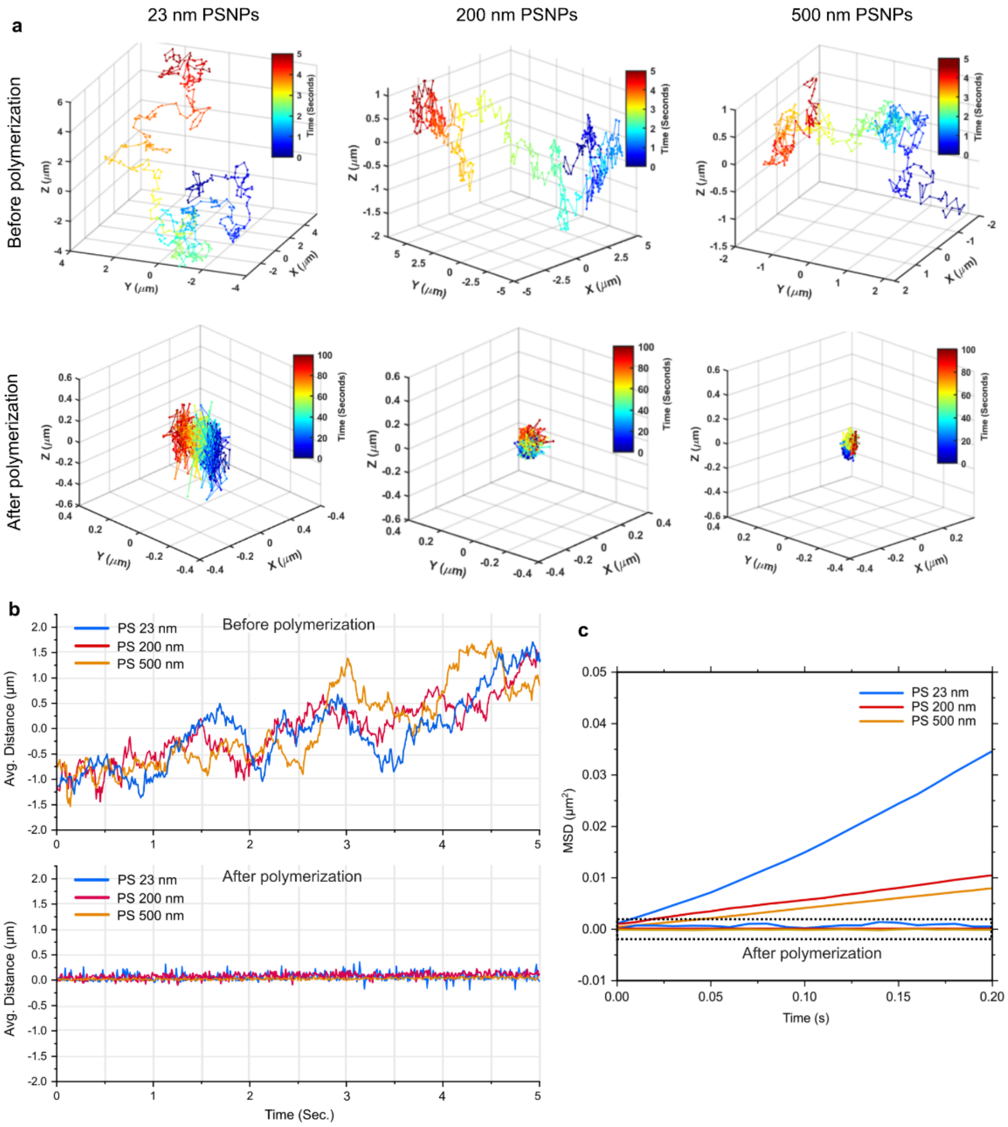

***S1.1*. Diffusion analysis of PSNPs before and after polymerization.** (a) Diffusion of PSNPs with diameters of 23, 200, and 500 nm, observed in the pre- and post-polymerization states. (b) Average distance versus time plots for the particles, derived from 3D diffusion data, showing comparative dynamics before and after the transition to a polymerized state. (c) Mean Squared Displacement (MSD) versus time plots for particles of varying sizes, correlating particle size with diffusion characteristics before and after polymerization.

The viscosity of the solution can be obtained from the Stokes-Einstein relation by using the diffusion coefficient value and is expressed by the equation:

$$D = \frac{K_B T}{6\pi\eta R_h}$$

The viscosity of the solution is calculated by implementing the value of the diffusion coefficient and the hydrodynamic radius (R_h) of the particle in the solution.

The 3D diffusion of PSNPs before and after photopolymerization is shown, with Figure S1.1(a) illustrating the diffusion of 23, 200, and 500 nm PSNPs in both pre- and post-photopolymerization states. The particle diffusion in the Polyacrylamide Photocuring Medium (PPM) solution was observed, and the derived diffusion coefficient indicates that the viscosity of the solution is 1.2 centipoise (cP), representing a 20% increase compared to water. To provide a detailed analysis, the average distance traveled over time is plotted for both pre- and post-photopolymerization states. The PSNPs display dynamic behavior in the PPM solution; however, post-photopolymerization, no movement is detected (Figure S1.1 b,c). Analysis of the diffusion coefficient as a function of time further confirms that PSNPs exhibit dynamic behavior before photopolymerization, with no diffusion occurring afterward. Notably, slight fluctuations are detected for the 23 nm PSNPs, likely attributable to experimental error, estimated to be approximately ±10 nm.

S1.2: Control experiment on localization precision after fixation

Fluorescent PSNPs with nominal diameters of 30, 60, and 200 nm were used to assess localization precision after fixation on glass and in the polyacrylamide (PAA) hydrogel. Particle fluctuations were examined under three conditions: (i) fixed on a glass coverslip and imaged with a 10 ms integration, (ii) embedded in PAA hydrogel and imaged with a 10 ms integration, and (iii) embedded in PAA and imaged with a 50 ms integration. Measurements were performed on the multiplane widefield setup using 488 nm excitation and 3D single-particle tracking.

For each particle size and condition, we determined the mean fluorescence intensity and the standard deviations of the localized positions along x, y, and z (Figure S1.2, Table S1). These standard deviations directly reflect the extent of fluctuations. As expected, the smallest particles show reduced fluorescence intensity in PAA hydrogel at 10 ms integration, and this intensity is largely recovered at 50 ms. For these small particles, positional fluctuations are already observed when fixed on glass and become slightly larger when embedded in PAA hydrogel at 10 ms, where the signal is lower; increasing the integration time to 50 ms restores the intensity and reduces the apparent motion. For 200 nm particles, the positional spreads remain smaller for fixed in glass and in PAA hydrogel with 50 ms integration, while the spread is higher in case of PAA hydrogel with 10 ms integration. The residual spreads in x, y, and z are similar for particles fixed on glass and in PAA hydrogel at comparable intensities. These results indicate that the small residual fluctuations observed for the smallest particles mainly reflect the limits of localization precision at low signal-to-noise, rather than incomplete fixation or diffusion inside the hydrogel.

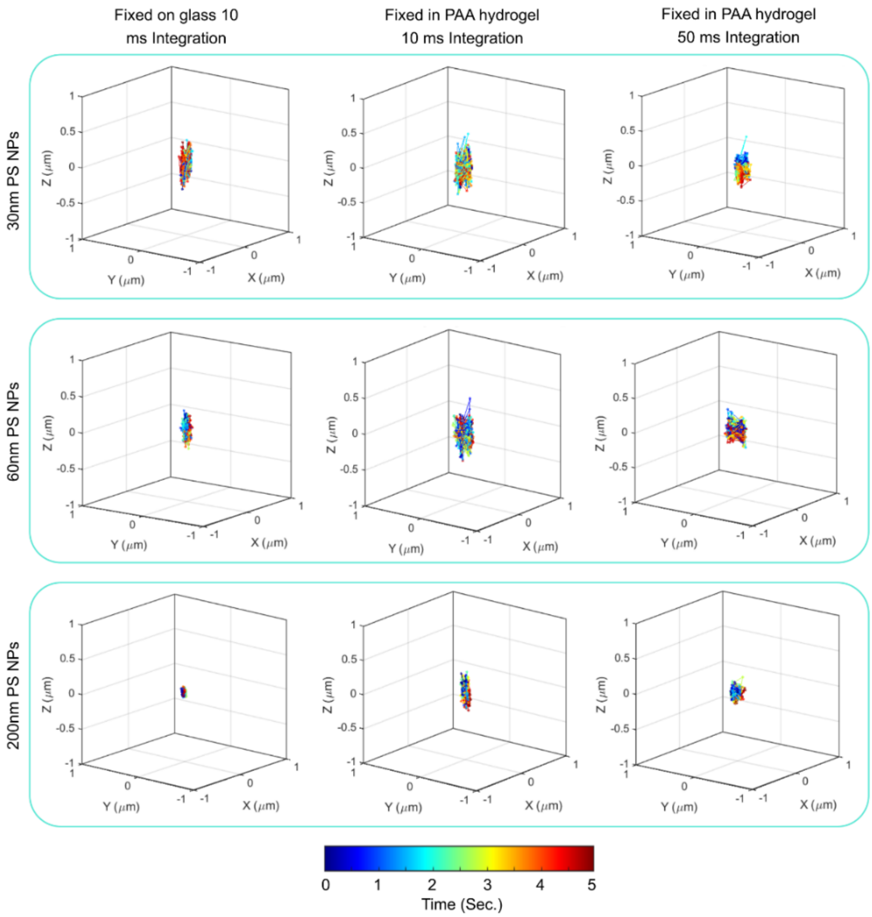

***S1.2*. Localization precision of fixed particles in glass and PAA hydrogel**. Single-particle tracking (SPT) experiments were performed on fluorescent PSNPs with diameters of 30, 60, and 200 nm under three conditions: particles fixed on a glass coverslip; particles embedded in PAA hydrogel and imaged with a 10 ms integration time; and particles embedded in PAA hydrogel and imaged with a 50 ms integration time.

Table S1. Mean fluorescence intensity and 3D positional standard deviations for fixed PSNPs under different conditions. For each particle size and condition, the mean intensity (a.u.) and σ_x, σ_y, σ_z (nm) were obtained from 3D single-particle tracking of fixed nanoparticles on glass and embedded in PAA hydrogel

Particle size (nm)	Condition	Mean intensity (a.u.)	σ_x (nm)	σ_y (nm)	σ_z (nm)
30	Glass, 10 ms	223.5 ± 29.7	24.19	19.73	84.85
30	PPM, 10 ms	132.7 ± 10.0	45.53	55.47	110.59
30	PPM, 50 ms	222.4 ± 16.8	35.56	42.08	93.39
60	Glass, 10 ms	161.1 ± 16.4	31.07	20.62	82.52

60	PPM, 10 ms	124.1 ± 9.4	47.64	61.69	121.14
60	PPM, 50 ms	157.9 ± 13.4	52.51	38.02	86.15
200	Glass, 10 ms	514.4 ± 41.5	15.29	11.31	28.07
200	PPM, 10 ms	184.8 ± 7.7	25.38	42.86	141.50
200	PPM, 50 ms	410.3 ± 34.1	23.20	34.78	58.14

Section S2: Control experiment for optically trapped assemblies in water and PPM

Assemblies of 1 µm PSMPs were formed with a 1064 nm linearly polarized laser at 1 W (after the objective) for 3 min in three different conditions: (i) water, (ii) water under UV irradiation, and (iii) the polyacrylamide photocuring medium (PPM). The assembly sizes were extracted from the lateral average intensity profiles after 3 minutes of irradiation (Figure S2). In all cases, assemblies were circular in shape and were around 20 µm in diameter. However, the mean assembly size in PPM is slightly smaller (by <1 µm) than in water (Figure S2c), consistent with the slightly higher viscosity of the PPM solution, which mildly slows particle approach to the trap leading to comparatively lower assembly size for the same fixed time, but does not change the overall assembly behavior. The control measurements show that assemblies formed in PPM closely match those formed in water, indicating that PPM does not limit the applicability of FRAME in these systems.

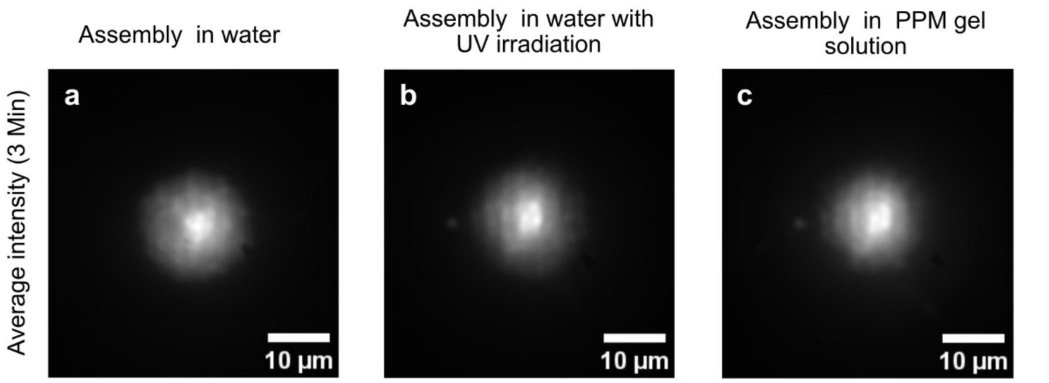

Figure S2**. Average intensity profiles of optically trapped assemblies in different media.** Assemblies of 1 µm PSMPs were formed with a 1064 nm laser at 1 W (after the objective) after 3 min of irradiation in (a) water, (b) water under UV irradiation, and (c) the PPM medium.

Section S3: Time evolution of optical matter formation with polystyrene microparticles

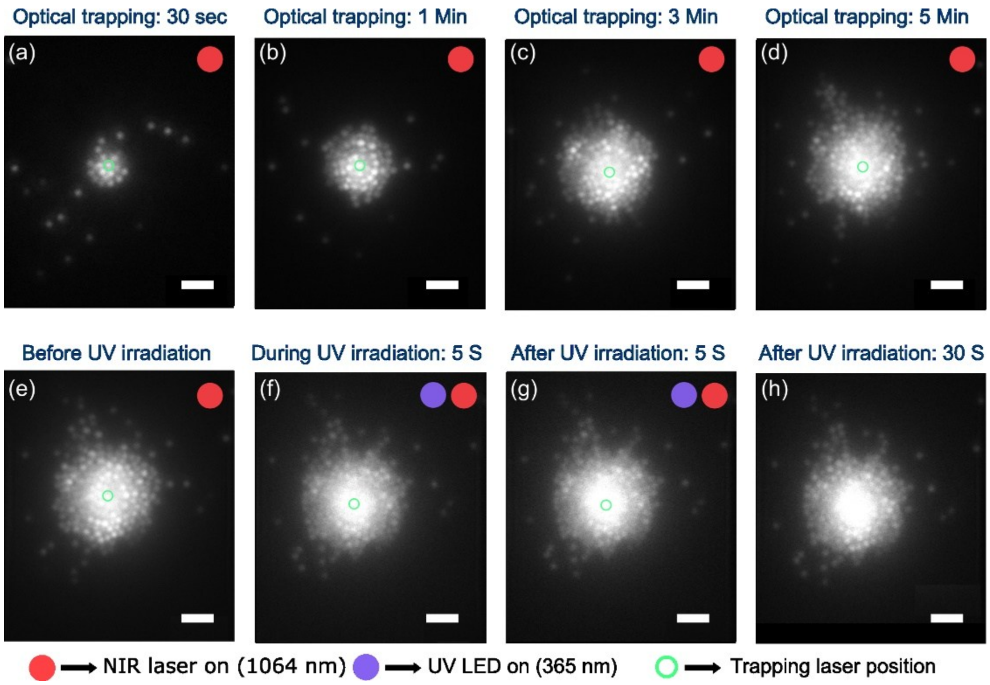

***Figure S3*. Time evolution of optical matter formation and permanent structure development in 1 µm diameter polystyrene microparticles.** (a-e) The microparticles, confined within an optical trap, evolve over a time span of 30 seconds to 5 minutes. The green circle at the center of each image marks the focal spot of the NIR trapping laser. The duration of NIR laser emission during optical matter formation is indicated by the red circle, while UV-LED irradiation is marked by a violet circle. (e-h) The transition from dynamic optical matter to permanent structures is depicted before, during, and after UV-LED irradiation. Scale bars: 5 µm.

Section S4: Quantitative structural validation of FRAME-fixed assemblies

S4.1: Confocal/SEM correlation

In this study, the segmentation of SEM images was conducted to accurately isolate individual particles and calculate their centroids for subsequent analysis. The process began with the conversion of the SEM images to grayscale, followed by cropping to the region of interest. The grayscale images were then enhanced using adaptive histogram equalization to improve the contrast, and a Gaussian filter was applied to smooth the images and reduce noise. Following this, edge detection was performed using the Laplacian of Gaussian (LoG) method, highlighting regions of rapid intensity change.

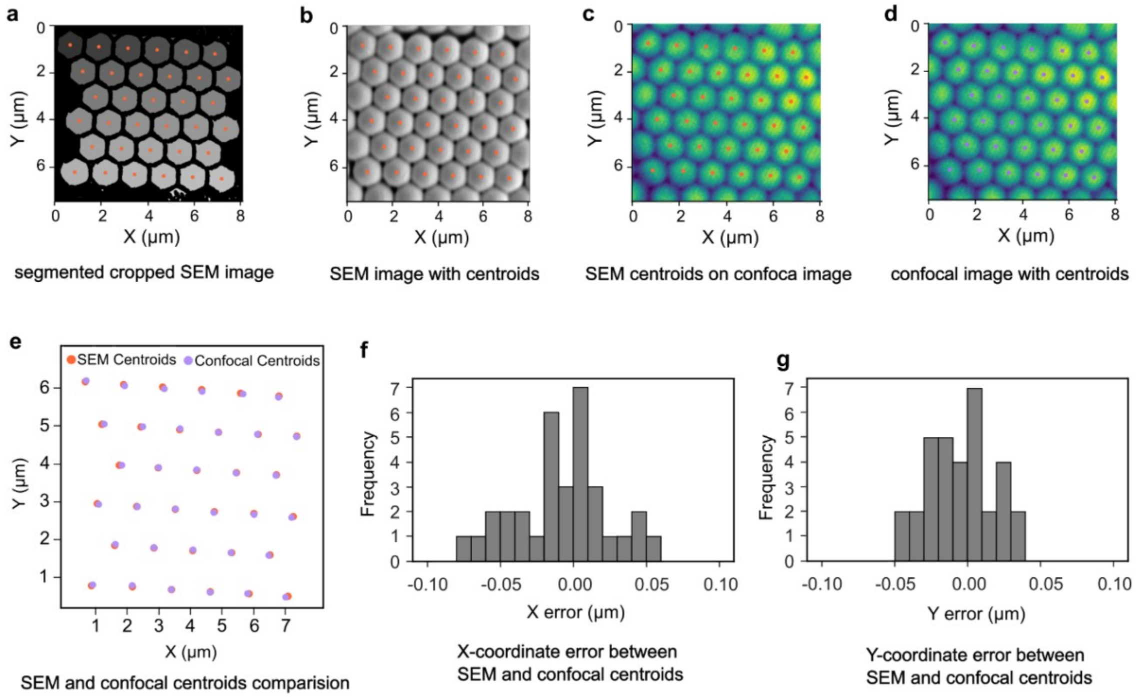

***Figure S4.1*. Correlation of confocal and SEM images.** (a, b) Centroids of the cropped region of the SEM image obtained through segmentation analysis. (c) Overlay of SEM centroids on the confocal image. (d) Confocal centroids obtained through 2D Gaussian fitting analysis. (e) The SEM and confocal centroids are shown in the figure, with position coordinates provided for all particles. (f, g) The position displacement error of confocal centroids from the SEM image along the X and Y axes, respectively.

To refine the segmentation, morphological operations including binary closing and dilation were used to connect disjointed edges and fill any small gaps within the detected particle boundaries. The centroids of these particles were calculated by measuring center of mass for each labeled particle based on its pixel distribution. For comparison with confocal microscopy data, these centroids were converted from pixel coordinates to micrometers using the specified pixel size (0.0172 μm per pixel).

Further analysis involved comparing these SEM-derived centroids with those obtained from confocal images. Confocal centroids were calculated using 2D Gaussian fitting to accurately determine the particle centers, accounting for any potential deviations in particle positioning (Figure S4.1). This comprehensive approach ensured precise calculation and comparison of particle centroids across different imaging modalities, facilitating detailed structural analysis of the colloidal assemblies.

S4.2: 3D correlation of FRAME-fixed assemblies before and after drying

To assess possible Z-axis distortions and anisotropic shrink/swell of the hydrogel during drying, we performed a 3D correlative registration of a single FRAME-fixed assembly of 1 μm PSMPs before and after drying for SEM preparation. The assembly was formed and fixed in PAA hydrogel as described in the main text. A first confocal 3D stack was acquired in the hydrated gel using 488 nm excitation. The sample was then dried at room temperature following the SEM drying protocol (including Au/Pd sputtering) while remaining immobilized on the coverslip. Finally, a second confocal 3D stack was recorded from the same field of view.

For each state ("before drying" and "after drying"), the 3D fluorescence volume was pre-processed by summing along the Z-axis to obtain an XY projection. Rigid registration (rotation + translation) of the XY projections was performed using a multimodal similarity metric following the MATLAB workflow "Register Multimodal 3-D Medical Images" (MathWorks documentation). In this approach, one projection is kept fixed and the other is treated as the moving image; the moving image is then rotated and translated until the two intensity patterns are optimally aligned, which is well suited to multimodal data where absolute intensities differ. This is followed by determination of the optimal Z shift by cross-correlation of the axial intensity profiles (Figure S4.2a-c). The resulting transformation was applied to the full 3D volume. Particle centroids were then detected in both images and matched using a sphere-gated iterative closest point (ICP) procedure, allowing only one-to-one correspondences within a fixed 3D search radius.

By 3D Gaussian fitting, 140 centroids were detected before drying and 137 after drying (maximum possible matches = 137). The ICP procedure yielded 121 matched particles. Overlays of the matched centroids in the XY, XZ and YZ planes show excellent agreement between the "before" and "after" configurations (Figure S4.2d-f). The lateral ($\sigma_{X,Y}$) and axial (σ_Z) deviations were only 40 and 87 nm, respectively, over an order of magnitude smaller than the particle size. Histograms of signed XY and Z errors are centered at zero, with no systematic bias in Z, indicating that any shrinkage during drying is minimal and essentially isotropic (Figure S4.2g,h). These results confirm that, for 1 μm PSMPs assemblies, drying and SEM preparation do not significantly distort (<100 nm) the 3D structure of the FRAME-fixed assemblies.

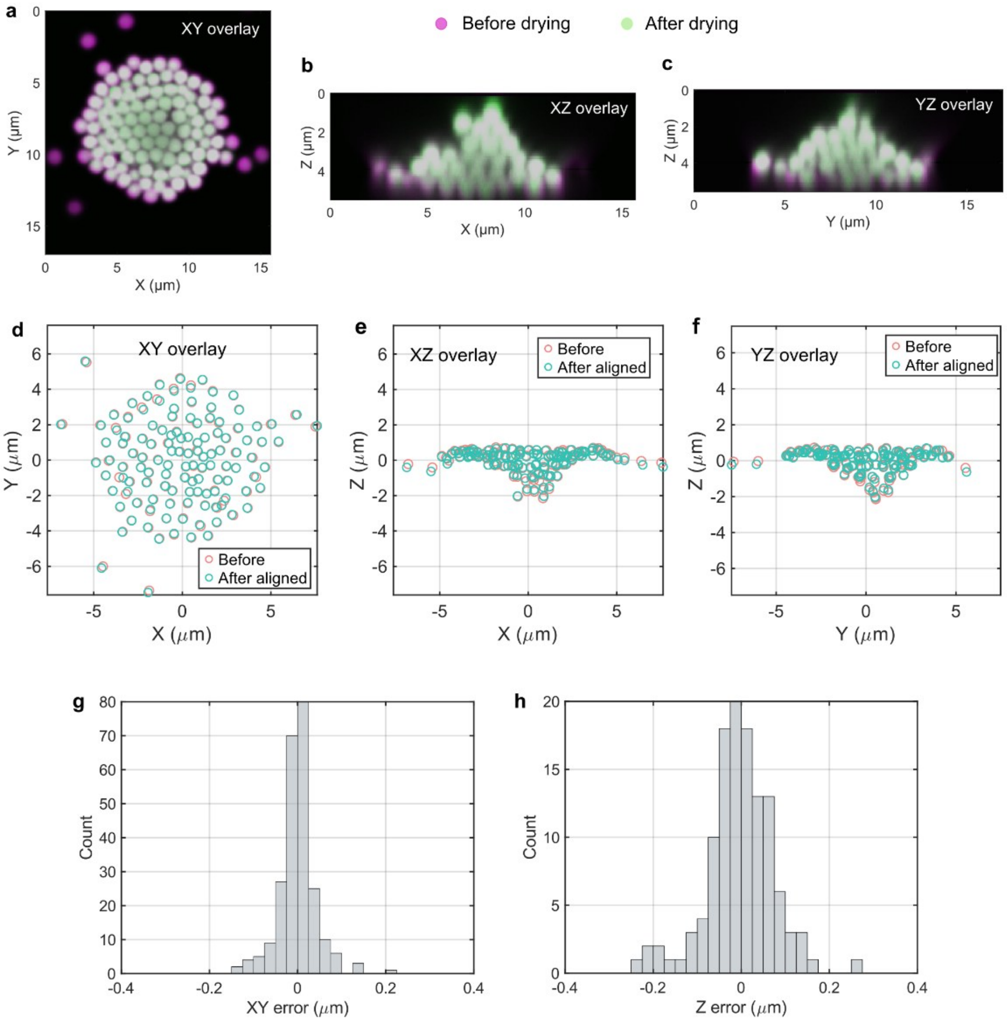

***Figure S4.2*. A 3D correlative analysis of a FRAME-fixed 1 µm PSMPs assembly before and after drying.** (a-c) Confocal overlays of the same assembly before (magenta) and after (green) drying in the XY, XZ and YZ planes, showing minimal change in overall shape. (d-f) Overlays of the matched particle centroids before (red circles) and after (green circles) rigid alignment in XY, XZ and YZ. (g,h) Histograms of the signed XY and Z errors for the 121 matched particles.

Section S5: 3D structural analysis of optical matter

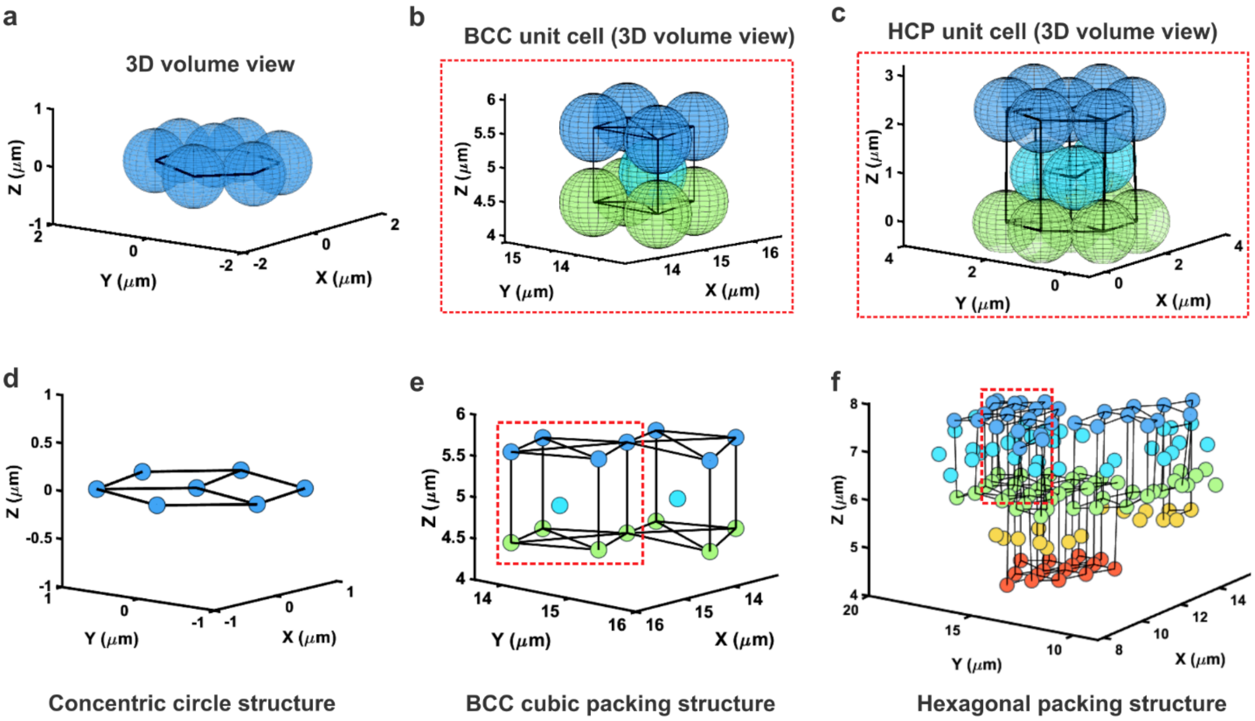

***Figure S5*. 3D structural analysis of optical matter formed at different Z-depths.** (a-c) 3D volumetric representation of concentric circle, body-centered cubic (BCC) and hexagonal close-packed (HCP) structure, respectively. (d-f) Structural analyses in 3D of concentric circular, BCC cubic packing, and hexagonal packing structures, from which the unit cells were derived.

Section S6: Measurement of beam waist diameter

The beam waist diameter $w(z)$ was calculated as a function of the axial position z using the Gaussian beam propagation formula,

$$w(z) = w_0\sqrt{1 + \left(\frac{z}{z_R}\right)^2}$$

Where Z_R is the Rayleigh range which defines the distance over which the beam maintains a relatively narrow width and given by, $Z_R = \frac{\pi w_0^2}{\lambda}$, with λ being the wavelength of the laser. The beam waist radius (w_0) is the value for the radius of the beam at focus. By computing w(z) across a range of positions from 0 to 5 µm, we obtained a detailed profile of the beam's expansion as it propagates away from the focus. From the backscattered intensity profile at the focal spot, we determined the beam waist diameter (w_0) to be approximately 1.8 µm, with a power density (I_0) of 19.6 MW/cm². The beam waist diameter at Z-depths of -1.5 µm, -2.5 µm, and -3.5 µm was calculated as 2.13 µm, 2.66 µm, and 3.15 µm, respectively, with corresponding power densities of 14 MW/cm², 8.97 MW/cm², and 6.41 MW/cm² (Figure S6).

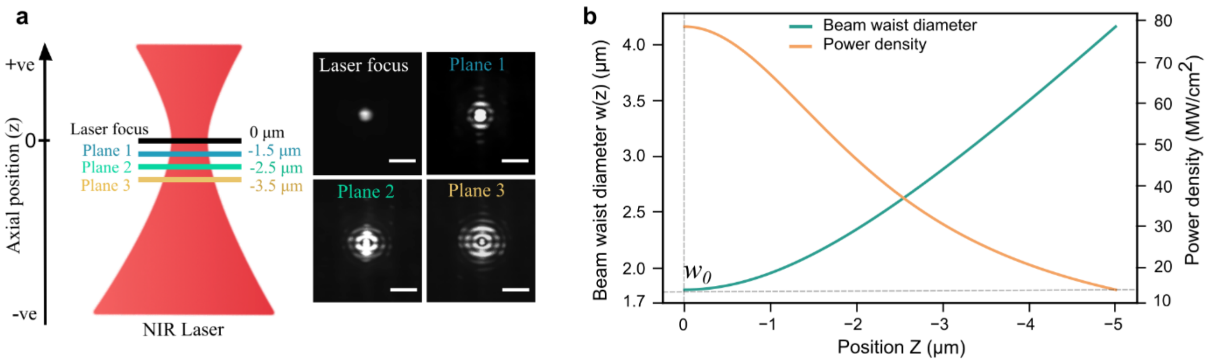

***Figure S6*. Beam waist diameter and power density vs. Z-depth position.** (a) Illustration of the simulated beam waist of the NIR trapping laser at varying focal depths, accompanied by the backscattered intensity profile obtained from the widefield multiplane setup at different focal depths. (b) The plot shows the beam waist diameter (µm) and power density (MW/cm²) as a function of position along the Z-axis (0-5 µm) for a laser with a 1.064 µm wavelength and 1.8 µm beam waist (w_0). The green line represents the beam waist diameter, and the orange line represents power density.

Section S7: 3D confocal image of optical matter with sub-diffraction limit nanoparticles

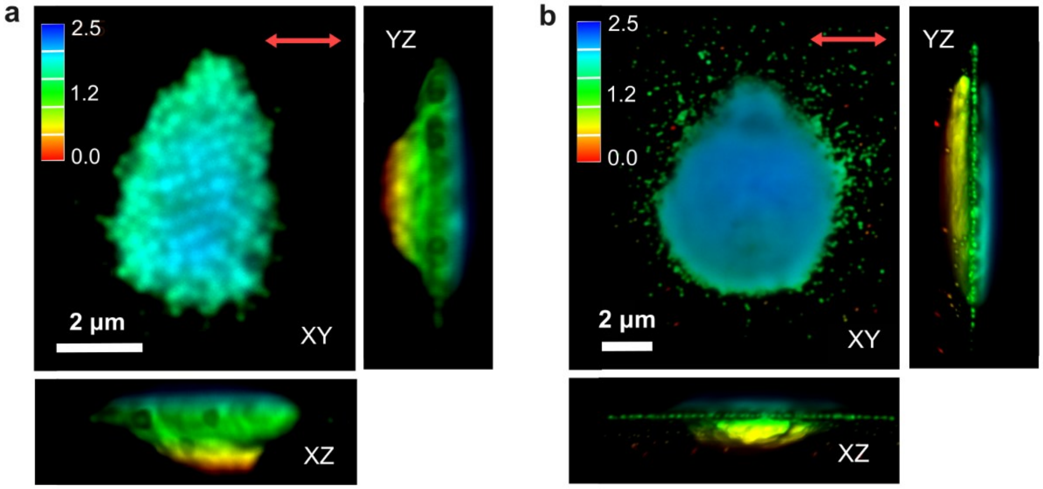

***Figure S7*. 3D confocal image of optical matter composed of sub-diffraction limit nanoparticles.** (a) 3D confocal microscopy image of optical matter composed of 300 nm polystyrene nanoparticles fixed in a hydrogel, with the red arrow indicating the polarization direction. (b) 3D confocal microscopy image of permanent optical matter composed of 200 nm polystyrene nanoparticles within a hydrogel matrix. Each scale bar represents 2 μm.

Section S8: 3D STED super-resolution structural analysis of FRAME-fixed 200 nm PSNPs assemblies

Assemblies of 200 nm fluorescent polystyrene NPs were formed by optical trapping, locked using FRAME, and imaged on a commercial Leica STED microscope (Leica TCS SP8X). STED imaging was performed with a 100×/1.40 oil-immersion objective using 633 nm excitation and a pulsed 775 nm depletion beam (80 MHz), as detailed in the Materials and Methods section. A 3D STED Z-stack was acquired and deconvolved, yielding super-resolved volumes in which individual nanoparticles are clearly visible throughout the assembly (Figure S8). Local intensity maxima in the deconvolved stack were fitted with 3D Gaussian point-spread functions to obtain sub-pixel centroid coordinates $(x_{i,}y_{i,}z_i)$ for each particle i. These centroids were converted to micrometers using the calibrated pixel sizes in XY (12 nm) and Z (150 nm), and used to generate the depth-color-coded 3D rendering in Figure S8a and the corresponding projections in Figure 6d,e.

To quantify the in-plane nearest-neighbor (NN) spacing, we used only the lateral coordinates $r_i = (x_i, y_i)$. For each particle i, we computed the Euclidean distance in the image plane to all other particles $j \neq i$,

$$d_{ij} = \|r_i - r_j\|_2 = \sqrt{(x_i - x_j)^2 + (y_i - y_j)^2},$$

and defined the NN distance as

$$d_i^{NN} = \min_{j \neq i} |r_i - r_j|$$

We evaluated the in-plane NN spacing from the centroid coordinates in the XY plane. The resulting NN center-to-center distances have a mean value of ≈177 nm, reflecting neighbor separations on the particle-diameter length scale. To visualize the spread, we plotted the signed NN distance deviations relative to the expected center-to-center spacing d_0 of 200 nm particles, $\Delta d_i = d_i^{NN} - d_0$. The corresponding histogram (Figure S8b) shows a distribution with a standard deviation of ≈86 nm. Distance smaller than d_0 (negative Δd_i) are expected in this system because the assembly is multilayered, so particles are not confined to a single plane, and the XY-projected nearest neighbors can appear closer than the nominal diameter. Overall, these values are consistent with a compact 3D, multilayer arrangement: because the particles are arranged across multiple layers rather than confined to a single plane. The NN can be found at different axial positions, which broadens the in-plane (XY) NN distribution and shifted the mean values below the nominal particle diameter compared with a single, perfectly close-packed monolayer.

Axial positions z_i from the same 3D Gaussian fits were binned to obtain particle counts as a function of depth (Figure S8c), revealing several well-defined layers across the assembly. To probe longer range in-plane order, we formed a z-sum projection of the deconvolved STED Z-stack and computed the magnitude of its Fourier transform. The resulting spectrum (Figure S8d) shows a diffuse ring rather than sharp Bragg peaks, consistent with short-range order and the absence of pronounced long-range crystallinity.

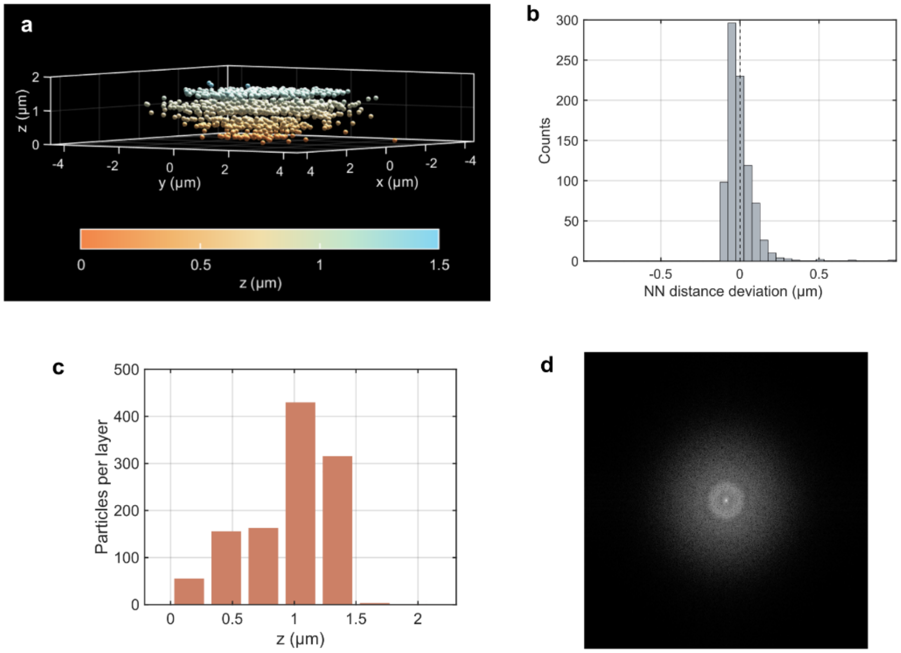

***Figure S8*. STED-based structural analysis of a FRAME-fixed assembly of 200 nm fluorescent nanoparticles.** (a) 3D rendering of particle centroids obtained from 3D Gaussian fitting of the deconvolved STED Z-stack, colour-coded by axial position z. (b) Histogram of nearest-neighbor distance deviations in the XY plane, showing a narrow distribution consistent with dense local packing. (c) Particle counts as a function of axial position, revealing a layered structure across the assembly. (d) Magnitude of the Fourier transform in the XY plane of the centroid-based z-sum projection, displaying a diffuse ring without sharp Bragg peaks, indicative of short-range order and the absence of pronounced long-range crystallinity.

Section S9: Dark-field scattering spectra of FRAME-fixed 1 µm PSMPs and 200 nm PSNPs assemblies

Dark-field scattering spectra were measured on FRAME-fixed assemblies of 1 µm PSMPs and 200 nm PSNPs using rectangular regions of interest (ROIs) at the center and edge of each assembly (Figure S9a). For each ROI, pixel intensities were averaged, a background spectrum from a nearby particle-free region was subtracted, and the resulting spectra were normalized (Figure S9b), covering the 515–850 nm range accessible with our current dark-field setup.

For 200 nm PSNPs, the spectra show a clear maximum around ~600 nm, consistent with Mie-type scattering, together with weaker features matching the yellow–green dye fluorescence band. The 1 µm PSMPs assemblies exhibit a strong broadband tail within this window, with higher intensity at the center than at the edge, consistent with denser packing. The main photonic resonance of the closely packed 1 µm assemblies is expected to extend further into the near-IR and is therefore not captured within the measured spectral range. Because the assemblies are immobilized by FRAME, they can in future be transferred to dedicated dark-field spectroscopy setups optimized for NIR detection, enabling measurements over a broader spectral range and a more detailed mapping of their metamaterial-relevant optical response.

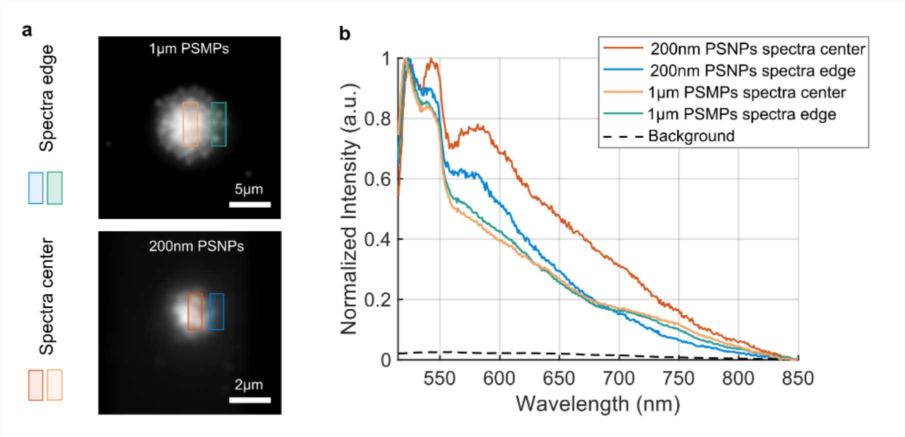

***Figure S9*. Dark-field scattering spectra of FRAME-fixed assemblies of 1 µm PSMPs and 200 nm PSNPs.** (a) Dark-field images with ROIs at the center and edge used for spectral extraction. (b) Background-subtracted spectra (515-850 nm), normalized at 500 nm, comparing center vs edge regions. Scale bars: 5 µm (top), 2 µm (bottom).

Section S10: Fixation of optical matter with Silica and gold nanoparticles using FRAME

This section demonstrates the applicability of FRAME for fixing optical assemblies composed of dielectric, and metallic nanoparticles under 1064 nm near-infrared laser illumination. Figure S10 shows widefield and SEM images of 1 µm silica assemblies formed in a 1064 nm optical trap at 1 W laser power (after the objective) over 5 minutes, with the SEM provides high-resolution image of the assembly after FRAME fixation. For the gold assemblies, the top row in Figure S10c presents bright-field images of 400 nm spherical gold nanoparticles forming optical-binding structures at the glass-solution interface under circular and linear polarization of a 1064 nm trapping beam at 25 mW (after the objective) over 5 minutes before fixation, whereas the bottom row shows the corresponding dark-field images of the assemblies after FRAME fixation.

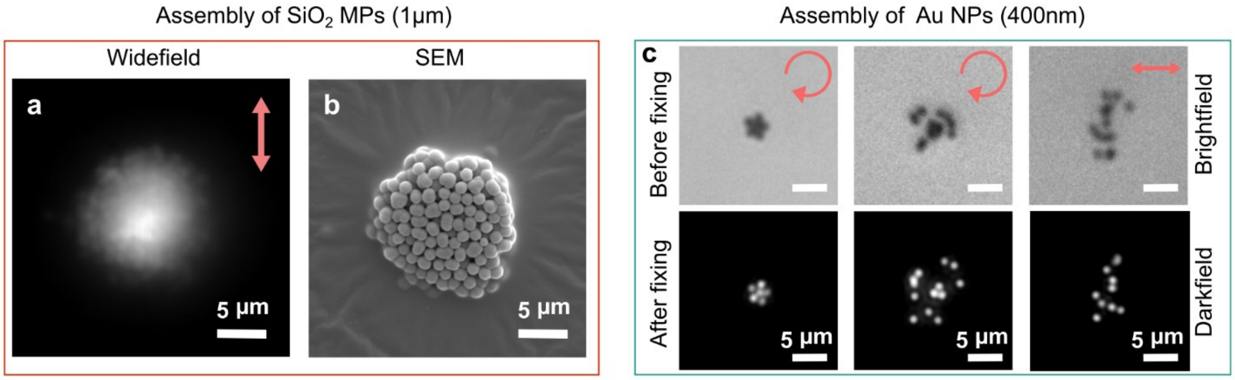

***Figure S10*. Fixation of silica and gold nanoparticle assemblies using FRAME.** (a) Widefield image of a 1 µm silica microparticle assembly formed in an optical trap at 1 W 1064 nm laser power (after the objective) before fixation. (b) Corresponding SEM image of the FRAME-fixed assembly, resolving individual particles and their packing. (c) Bright-field images of 400 nm spherical gold nanoparticle assemblies at the glass–solution interface before fixation (25 mW after the objective) under circularly (first two columns) and linearly (third column) polarized 1064 nm trapping light; the corresponding dark-field images after FRAME fixing are shown below. Red arrows indicate the polarization direction or type. Each scale bar represents 5 µm.

Section S11: Long-term stability of FRAME-fixed assemblies under ambient storage

To assess long-term structural stability, we re-imaged the same FRAME-fixed assembly of 1 μm PSMPs by SEM 528 days after fixation, following storage in a closed box under normal laboratory ambient conditions (room temperature, ambient humidity). The two SEM images were aligned, and particle positions were compared by measuring their radial distances from the assembly center along predefined transects in the "before" and "after" images. The mean radial shift was < 20 nm per 1 μm radial distance (< 2% of the particle diameter), which is within the uncertainty of the imaging and alignment procedure and indicates no measurable drift, dilation, or anisotropic distortion over ~1.5 years.

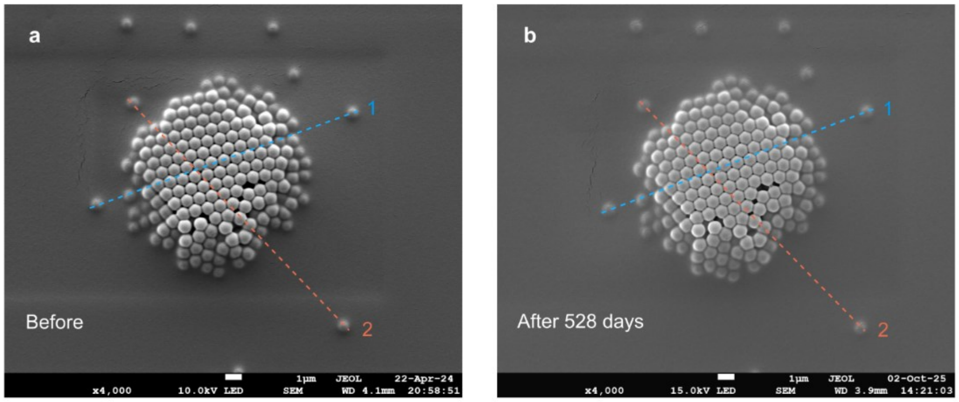

Figure S11. **Long-term ambient stability of a FRAME-fixed 1 μm PSMPs assembly.** (a) SEM image acquired shortly after fixation. (b) SEM image of the same assembly after 528 days stored in a closed box under normal laboratory ambient conditions (room temperature, ambient humidity). Dashed lines (1, 2) indicate radial directions used to compare particle positions. Scale bars: 1 μm.

Section S12: Widefield experimental setup with integrated optical trap

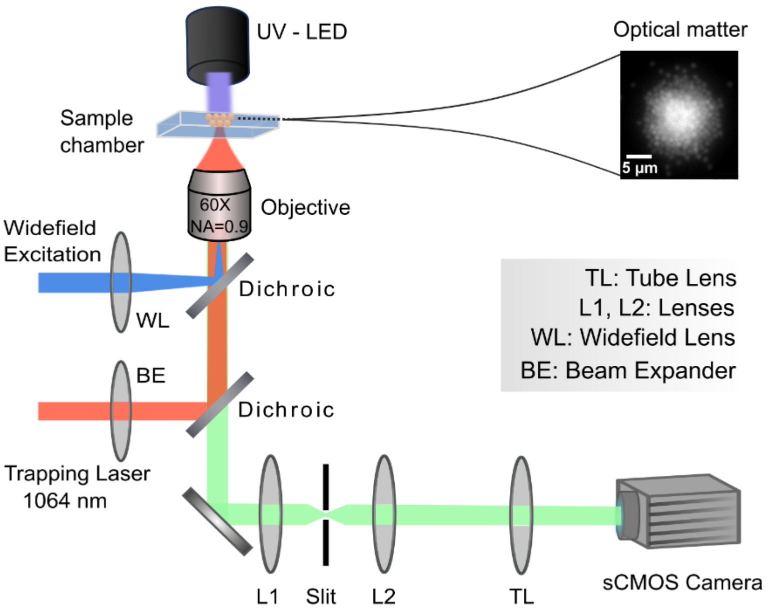

***Figure S12*. Optical trapping setup.** This figure illustrates the schematic of the widefield experimental setup used for optical trapping experiments. The setup includes a widefield microscope equipped with an excitation source and a near-infrared (NIR) trapping laser, along with a specialized sCMOS camera for image acquisition. The UV-LED source is positioned above the objective lens to facilitate photopolymerization.

Section S13: Power-dependent radical formation in LAP under UV-365 irradiation

The power-dependent radical formation of the LAP photoinitiator was investigated by monitoring the absorption spectra over time. Upon absorbing a photon, the chromophore within the LAP molecule undergoes a molecular transformation, leading to the formation of radicals (Figure S13). To quantify this process, the absorption spectra of the LAP photoinitiator were recorded using a UV-Vis spectrophotometer (Agilent Cary 60 UV-Vis Spectrophotometer). The sample was subjected to UV-365 irradiation from a focused LED at various power densities and broad absorption band of the molecule was observed in the wavelength range of 350-400 nm.

Spectral measurements were taken at time intervals of 15 seconds, 30 seconds, 1 minute, and 2 minutes, corresponding to irradiation power densities of 4, 10, 15, 25, and 50 mW/cm², respectively. These measurements allowed us to observe the kinetics of radical formation and how it is influenced by both the power density of the UV irradiation and the duration of exposure. It can be observed that, with the irradiation of VU-LED at 25 mW/cm² the absorption band saturates, which indicates the fast formation of radicals at this irradiation power.

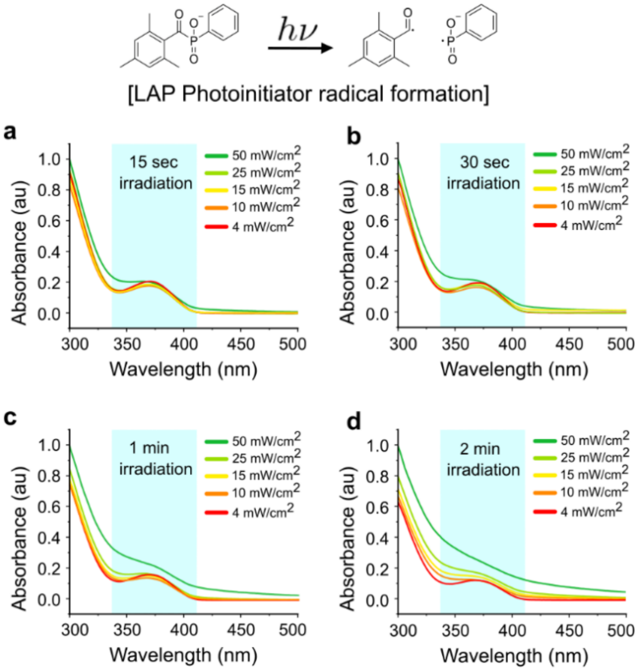

Figure S13. **Power dependent radical formation of LAP photoinitiator.** Radical formation in the LAP photoinitiator is observed upon UV LED irradiation at power densities of 4, 10, 15, 25, and 50 mW/cm². Panels (a) through (d) illustrate changes in the absorbance of the LAP photoinitiator over time intervals of 15 seconds, 30 seconds, 1 minute, and 2 minutes, respectively, under UV-365 irradiation at varying power levels.

Section S14: Widefield-multiplane experimental setup

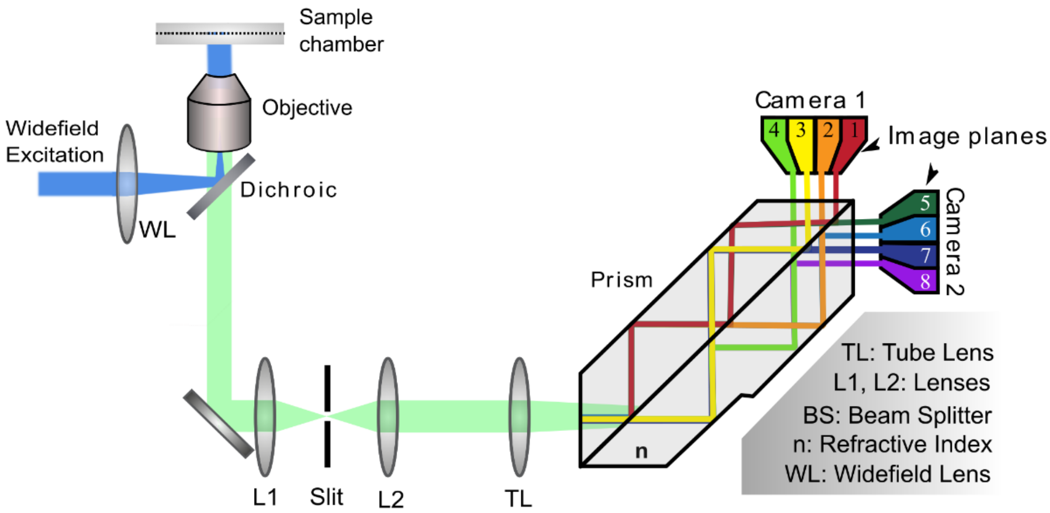

***Figure S14*. Widefield-multiplane experimental setup.** This figure illustrates the schematic of the multiplane widefield experimental setup used for the single-particle tracking (SPT) experiments. The setup comprises a widefield microscope equipped with an excitation source and a specialized prism containing mirrors and beamsplitters positioned before the camera to create eight planes at different Z-depths.

Section S15: FRAME fixation of PSMPs formed at different trapping wavelengths and polarizations

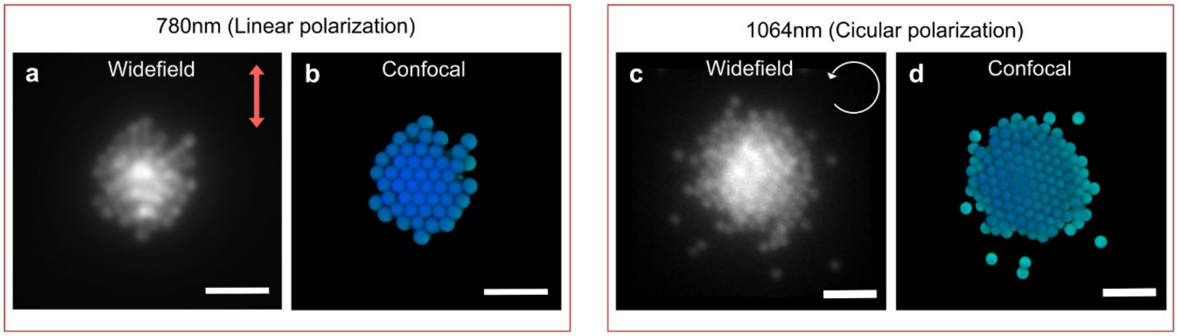

***Figure S15*. Optical matter assemblies of 1 μm PSMPs formed under different trapping conditions and fixed by FRAME.** (a,b) Widefield and confocal image of an assembly of 1 μm PSMPs formed with a 780 nm linearly polarized optical trap. (c,d) Widefield and confocal image of an assembly of 1 μm PSMPs formed with a 1064 nm circularly polarized optical trap. Scale bars: 5 μm.

Section S16: Fixation of optical matter by FRAME (Supplementary Movie S1)

This movie (Movie S1) validates the FRAME protocol for the fixation of optically assembled matter. The optical matter was formed by trapping 1 μm polystyrene particles using a 1064 nm laser at 1 W power (post-objective). The video demonstrates the entire process before, during, and after photo-irradiation highlighting the transition from dynamic assemblies to fixed structures. Fixation was achieved through the FRAME method.
